# Efficacy and safety of newer P2Y_12_ inhibitors for acute coronary syndrome: a network meta-analysis

**DOI:** 10.1038/s41598-020-73871-x

**Published:** 2020-10-08

**Authors:** Yue Fei, Cheuk Kiu Lam, Bernard Man Yung Cheung

**Affiliations:** 1 Division of Clinical Pharmacology and Therapeutics, Department of Medicine, Queen Mary Hospital, The University of Hong Kong, Pokfulam, 102 Pokfulam Road, Hong Kong, China; 2 State Key Laboratory of Pharmaceutical Biotechnology, The University of Hong Kong, Pokfulam, Hong Kong, China; 3 Institute of Cardiovascular Science and Medicine, The University of Hong Kong, Pokfulam, Hong Kong, China

**Keywords:** Acute coronary syndromes, Drug therapy, Medical research

## Abstract

Whether newer P2Y_12_ inhibitors are more efficacious and safer than clopidogrel and whether there is a superior one remain uncertain. We compared the effect of P2Y_12_ inhibitors on clinical outcomes in patients with acute coronary syndrome (ACS). Randomized controlled trials comparing clopidogrel, prasugrel, ticagrelor, or cangrelor, in combination with aspirin were searched. Sixteen trials with altogether 77,896 patients were included. Compared to clopidogrel, cardiovascular mortality was reduced with prasugrel (OR 0.85, 95% CI 0.75–0.97) and ticagrelor (0.82, 0.73–0.93). Myocardial infarction (0.75, 0.63–0.89) and major adverse cardiovascular events (0.80, 0.69–0.94) were reduced by prasugrel. Stent thrombosis was reduced by prasugrel (0.49, 0.38–0.63), ticagrelor (0.72, 0.57–0.90), and cangrelor (0.59, 0.43–0.81). It was reduced more by prasugrel than ticagrelor (0.69, 0.51–0.93). There were more major bleeds with prasugrel (1.24, 1.05–1.48). Thrombolysis in Myocardial Infarction (TIMI) major bleeding was increased with prasugrel compared to clopidogrel (1.36, 1.11–1.66) and ticagrelor (1.33, 1.06–1.67). TIMI minor bleeding was increased with prasugrel (1.44, 1.16–1.77) and cangrelor (1.47, 1.01–2.16) compared to clopidogrel while it was increased with prasugrel compared to ticagrelor (1.32, 1.01–1.72). Prasugrel is preferable to those ACS patients at low bleeding risk to reduce cardiovascular events whereas ticagrelor is a relatively safe antiplatelet drug of choice for most patients.

## Introduction

Clopidogrel is a commonly used P2Y_12_ inhibitor recommended for the standard treatment and secondary prevention of ischemic events in acute coronary syndrome (ACS) patients^1,2^. As a prodrug, its limitations such as low bioavailability, delayed onset of action, variability in patient responsiveness, and irreversible antiplatelet effect^3–5^ led to the development of more potent, newer P2Y_12_ inhibitors. Prasugrel, a third-generation thienopyridine with irreversible inhibition of P2Y_12_ receptors, has been observed to provide a better clinical efficacy than clopidogrel, but at the expense of an increased risk of major bleeding^6^. Ticagrelor, a cyclopentyl triazolo-pyrimidine, is a direct-acting and reversible P2Y_12_ inhibitor^7–9^ showing benefits in reducing ischemic events, which makes it a promising option for the treatment of ACS patients. These advantages, however, are accompanied by a numerically higher risk of major bleeding^8^. Cangrelor is an intravenous, fast- and direct-acting blocker of P2Y_12_ receptor^10–13^. It was reported to reduce ischemic events, especially myocardial infarction (MI) and stent thrombosis, without a significant increase in severe bleeding^13,14^.

The efficacy and safety of newer P2Y_12_ inhibitors in ACS influenced the recommendations in the clinical guidelines. It must be recognized that the greater and more rapid inhibition of platelet aggregation may be counterbalanced by the higher risk of bleeding complications^6,8,15^. In the 2016 American College of Cardiology (ACC)/American Heart Association (AHA) guideline on dual antiplatelet therapy (DAPT), ticagrelor was recommended over clopidogrel as a maintenance therapy in patients with non-ST-segment elevation (NSTE)-ACS or ST-segment elevation myocardial infarction (STEMI) receiving DAPT after percutaneous coronary intervention (PCI), while prasugrel was recommended for those patients at low bleeding risk and without prior stroke or transient ischemic attack (Class IIa)^16^. The 2017 European Society of Cardiology (ESC)/European Association for Cardio-Thoracic Surgery (EACTS) guideline recommended ticagrelor for ACS patients at moderate to high ischemic risk regardless of the initial treatment, but prasugrel for ACS patients undergoing PCI if there is no excess fatal bleeding or other contraindications (Class I)^17^.

Although there have been several randomized controlled trials (RCTs) comparing the efficacy and safety of newer P2Y_12_ inhibitors with that of clopidogrel in patients with ACS, there were no head-to-head trials between newer P2Y_12_ inhibitors until the report of the two recent trials directly comparing prasugrel with ticagrelor^18,19^. It is necessary to update the efficacy and safety profiles of P2Y_12_ inhibitors in ACS patients, particularly to assess the comparative effectiveness among newer P2Y_12_ inhibitors. Because direct evidence is limited and inadequately powered, indirect comparisons among newer P2Y_12_ inhibitors should also be sought as supportive evidence. Therefore, we performed a network meta-analysis to compare the effect of P2Y_12_ inhibitors on cardiovascular and bleeding events in patients with ACS in order to optimize therapy in clinical practice.

## Methods

### Search strategy and study selection

This network meta-analysis conforms with the reporting standards in the PRISMA statement. We searched MEDLINE, EMBASE, the Cochrane database, and ClinicalTrials.gov up to December 31, 2019 for RCTs using the terms “clopidogrel” or “prasugrel” or “ticagrelor” or “cangrelor” or “P2Y_12_ inhibitors” or “thienopyridine” or “antiplatelet therapy” or “DAPT” or “acute coronary syndrome” or “non-ST-elevation myocardial infarction” or “unstable angina”, and their synonyms and related keywords. Study inclusion criteria for this network meta-analysis were: (1) RCTs; (2) sample size of over 100 patients in total; (3) patients over 18 years of age with ACS; (4) allocation of different P2Y_12_ inhibitors in patients receiving DAPT; (5) reporting the number of cardiovascular events, bleeding events, and deaths. No language restrictions were enforced.

### Data extraction

Literature review and inclusion were carried out by two investigators (YF and CKL) independently. Disagreements were resolved by consensus. For eligible studies, information about the year of publication, sample size, the maximum duration of follow-up, loading dose and maintenance dose in each treatment arm, and patient characteristics including age, gender, body mass index, ethnic group, smoking status, baseline comorbidities of cardiovascular disease were extracted. Risk of bias was assessed using the Cochrane risk of bias assessment tool. The following components were evaluated: sequence generation, allocation concealment, blinding of participants and personnel, blinding of outcome, incomplete outcome data, and free from other bias. Each component was scored as a low, high, and unclear risk of bias.

### Study outcomes

The primary outcomes were cardiovascular death, MI, and stroke. Secondary outcomes were major adverse cardiovascular events (MACE) defined as a composite of cardiovascular death, MI, and stroke, definite or probable stent thrombosis, all-cause mortality, Thrombolysis In Myocardial Infarction (TIMI) major bleeding which included non-coronary artery bypass grafting (CABG)-related and CABG-related TIMI major bleeding, TIMI minor bleeding, and all major bleeding including TIMI major bleeding, PLATO (PLATelet inhibition and patient Outcomes)-defined major bleeding^8^ or Bleeding Academic Research Consortium (BARC) major bleeding (type 3, 4, or 5)^20^. For those trials reporting major bleeding with more than one definition, the priority selection criteria for analysis in all major bleeding was TIMI major bleeding over BARC major bleeding over PLATO-defined major bleeding.

### Statistical analysis

The efficacy and safety of different P2Y_12_ inhibitors were compared at the trial-level using a frequentist approach^21^. Pooled random-effects odds ratios (ORs) and 95% confidence intervals (95% CIs) were the summary statistics used. A 95% CI not including 1.00 or a p-value (two-tailed) less than 0.05 was considered statistically significant. Forest plots using random- and fixed-effects models to compare relative treatment effects were generated using the statistical package netmeta version 0.9-8 (https://cran.r-project.org/web/packages/netmeta/index.html) in R version 3.3.3. P-score was computed to determine the likelihood of the P2Y_12_ inhibitors being the best for protecting against each outcome. Subgroup analyses were performed for oral P2Y_12_ inhibitors and intravenous cangrelor (vs. clopidogrel), respectively.

Loop-specific approach was used to appraise the inconsistency between estimates derived from direct and indirect evidence in the network. τ^2^ estimate with values of 0.04, 0.14, and 0.40 corresponded to a low, moderate and high degree of heterogeneity, respectively. I^2^ statistics were used to assess the presence of heterogeneity within each pairwise comparison with I^2^ < 25%, within 25–50%, and > 50% corresponding to mild, moderate, and severe heterogeneity, respectively. Small study effects or potential publication bias was assessed visually by funnel plots and trim-and-fill test. Egger’s test for asymmetry in funnel plots would be performed only in those direct comparison groups having ten or more studies in case of misleading results.

Random-effects Bayesian framework^22^ with non-informative priors was used to perform sensitivity analysis in order to check the robustness of the study findings. In addition, the consistency of inferential estimates from hierarchical modelling was evaluated with a Bayesian framework by means of Markov chain Monte Carlo simulations in order to be similar to the frequentist estimates, and these were performed with 1000 tuning iterations and 5000 simulation iterations using R statistical package gemtc version 0.8-2 (https://cran.r-project.org/web/packages/gemtc/index.html) and rjags version 4-6 (https://cran.r-project.org/web/packages/rjags/index.html) to minimize Monte Carlo error. The protocol for this network meta-analysis was registered with the PROSPERO registry (number CRD42019122170).

## Results

**Figure 1:**
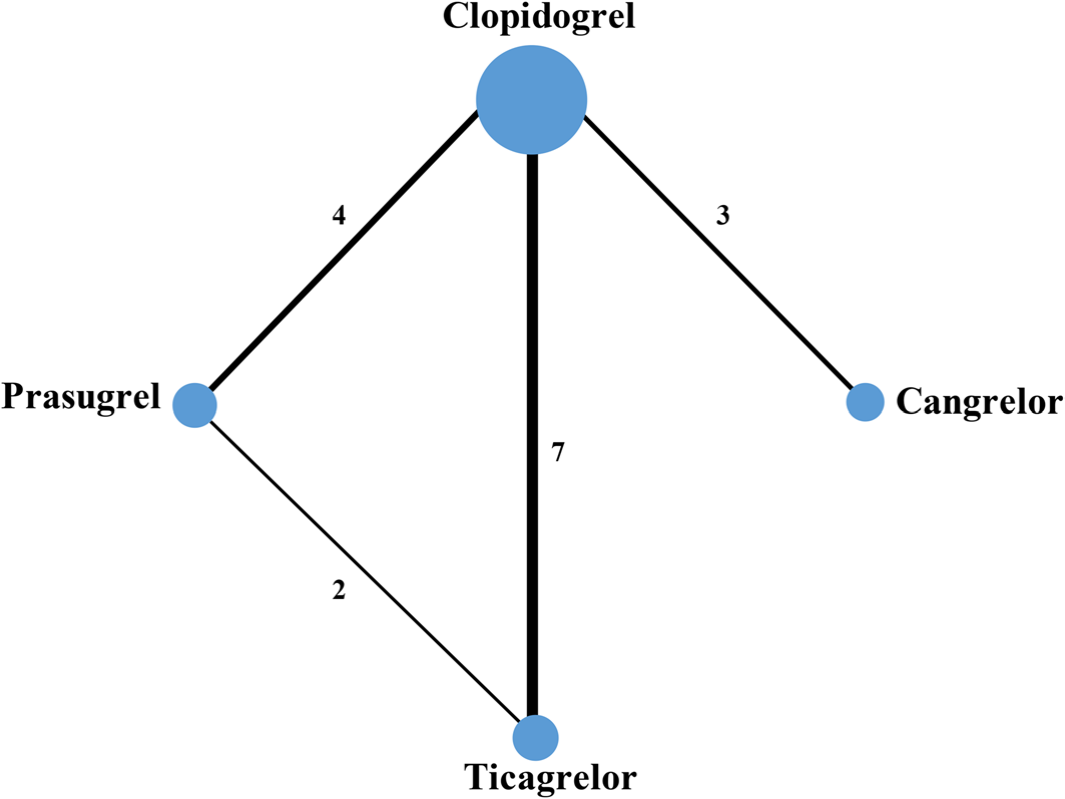
Network profile for the studies comparing different P2Y_12_ inhibitors involved in DAPT. Each line represents a pair of direct comparison between different P2Y_12_ inhibitors. The width of the lines is proportional to the number of trials comparing every pair of treatments, and the size of every circle is proportional to the number of randomly assigned participants (sample size).

A summary of the screening and selection process is described in the PRISMA flowchart (Supplementary Fig. S1). Twenty trials fulfilled the inclusion criteria^6,8,10,11,13,15,18,19,23–34^. However, doubling loading or maintenance dose of clopidogrel was used in the Thrombocytes And IndividuaLization of ORal antiplatelet therapy in percutaneous coronary intervention (TAILOR)^32^ and Xiong et al.^33^ trials; two doses of ticagrelor therapy rather than different P2Y_12_ inhibitors were compared in the Prevention of Cardiovascular Events in Patients with Prior Heart Attack Using Ticagrelor Compared to Placebo on a Background of Aspirin–Thrombolysis In Myocardial Infarction 54 (PEGASUS-TIMI 54) trial^15^; ticagrelor was compared with placebo rather than the other P2Y_12_ inhibitors in the Effect of Ticagrelor on Health Outcomes in Diabetes Mellitus Patients Intervention Study (THEMIS) trial^34^, so these trials were excluded. Sixteen two-armed trials with altogether 77,896 patients were eligible for this network meta-analysis^6,8,10,11,13,18,19,23–31^. There are seven trials comparing ticagrelor vs. clopidogrel (n = 24,629)^8,23–28^, four trials comparing prasugrel vs. clopidogrel (n = 23,118)^6,29–31^, three trials comparing cangrelor vs. clopidogrel (n = 24,901)^10,11,13^, two trials comparing ticagrelor vs. prasugrel (n = 5248)^18,19^. In total, 41,844 patients were randomized to newer P2Y_12_ inhibitors while 36,052 patients were randomized to clopidogrel. The loading dose of clopidogrel used in the comparison between ticagrelor vs. clopidogrel and prasugrel vs. clopidogrel was 300 or 600 mg, followed by 75 mg clopidogrel for maintaining treatment. Cangrelor was administered intravenously as a bolus (30 μg/kg of cangrelor or matching placebo), followed by an infusion (4 μg/kg per min of cangrelor or matching placebo), while 600 mg clopidogrel was given at the end of the infusion in the cangrelor groups; 300–600 mg clopidogrel was given to the comparator groups with different timings in the three cangrelor trials included^10,11,13^. For each outcome of interest, there were six theoretical comparisons (Fig. 1).

The main characteristics of the included trials are shown in Table 1. They all had a low risk of bias assessed using the components recommended by the Cochrane Collaboration (Supplementary Table S1 and Table S2 online). Baseline characteristics of patients enrolled in the sixteen trials are summarized in Supplementary Table S3 online. All included trials reported the frequencies of MACE, MI, and all-cause mortality. Fourteen trials reported the frequency of stroke^6,8,10,11,18,19,23–25,27–31^; 13 trials reported cardiovascular mortality^6,8,13,18,19,23–28,30,31^; nine trials reported the frequency of definite or probable stent thrombosis^6,8,10,12,13,18,19,25,31^. All the sixteen trials reported major bleeding events. Twelve trials reported TIMI major bleeding^6,8,10,12,13,18,23,25,28–31^ while three trials reported CABG-related TIMI major bleeding^6,8,28^ and seven trials reported non-CABG-related TIMI major bleeding^6,8,13,28–31^. Eleven trials reported TIMI minor bleeding^6,8,10,11,13,23,25,28–31^. Four trials reported BARC major bleeding^18,19,26,28^, in which the ISAR-REACT 5^19^ and the Safety and the Efficacy of Ticagrelor for Coronary Stenting Post Thrombolysis (SETFAST)^26^ trials reported BARC bleeding only without reporting TIMI bleeding. Four trials reported PLATO-defined major bleeding^8,24,27,28^ in which the Phase the International Study of Ticagrelor and Clinical Outcomes in Asian ACS Patients (PHILO)^24^ and Wang et al.^27^. trials did not report TIMI major bleeding. It should be noted that the definition of MACE varied across different trials (Supplementary Table S4 online). The definition of this composite outcome used in this network meta-analysis was the same as that in the original trial.

**Table 1:** Major characteristics of trials included in the network meta-analysis.

Studies	Year	ClinicalTrials.gov identifier	Number of patients	P2Y_12_ inhibitors	Primary endpoints	Definition of bleeding
DISPERSE-2^23^	2007	NA	661	Ticagrelor (AZD6140) vs. clopidogrel	TIMI major or minor bleeding	TIMI
PLATO^8^	2012	NCT00391872	18,624	Ticagrelor (AZD6140) vs. clopidogrel	Composite of death from vascular causes, myocardial infarction, or stroke	PLATO-defined^a^, TIMI
PHILO^24^	2015	NCT01294462	801	Ticagrelor vs. clopidogrel	Composite of death from vascular causes, myocardial infarction, or stroke	PLATO-defined^a^
Tang et al.^25^	2016	NA	400	Ticagrelor vs. clopidogrel	Composite of overall death, myocardial infarction, unplanned revascularization, and stroke	TIMI
SETFAST^26^	2017	NCT01930591	144	Ticagrelor vs. clopidogrel	Bleeding	BARC
Wang et al.^27^	2016	NA	200	Ticagrelor vs. clopidogrel	Composite of myocardial infarction, stroke, or cardiovascular death	PLATO-defined^a^
TREAT^28^	2018	NCT02298088	3799	Ticagrelor vs. clopidogrel	Major bleeding	TIMI, PLATO-defined^a^, BARC
JUMBO–TIMI 26^29^	2005	NA	904	Prasugrel vs. clopidogrel	Composite of TIMI major and minor hemorrhage	TIMI
TRILOGY ACS^30^	2012	NCT00699998	7243	Prasugrel vs. clopidogrel	Composite of death from cardiovascular causes, non-fatal myocardial infarction, or non-fatal stroke	TIMI, GUSTO
TRITON-TIMI 38^6^	2009	NCT00097591	13,608	Prasugrel vs. clopidogrel	Composite of cardiovascular death, non-fatal myocardial infarction, or non-fatal stroke	TIMI
PRASFIT-ACS^31^	2014	NA	1363	Prasugrel vs. clopidogrel	Composite of cardiovascular death, non-fatal myocardial infarction, and non-fatal ischemic stroke	TIMI
CHAMPION PCI^10^	2009	NCT00305162	8667	Cangrelor vs. Clopidogrel	Composite of death from any cause, myocardial infarction, or ischemia-driven revascularization at 48 h	GUSTO, TIMI, ACUITY
CHAMPION PLATFORM^11^	2009	NCT00385138	5295	Cangrelor vs. clopidogrel	Composite of death, myocardial infarction, or ischemia-driven revascularization 48 h after percutaneous coronary intervention	TIMI, GUSTO, ACUITY
CHAMPION PHOENIX^13^	2013	NCT01156571	10,939	Cangrelor vs. clopidogrel	Composite rate of death from any cause, myocardial infarction, ischemia-driven revascularization, or stent thrombosis in the 48 h	GUSTO, TIMI
PRAGUE-18^18^	2017	NCT02808767	1230	Ticagrelor vs. prasugrel	Composite of cardiovascular death, non-fatal myocardial infarction, or stroke	TIMI, BARC
ISAR-REACT 5^19^	2019	NCT01944800	4018	Ticagrelor vs. prasugrel	Composite of death, myocardial infarction, or stroke	BARC

*BARC* Bleeding Academic Research Consortium, *GUSTO* global utilization of streptokinase and tissue plasminogen activator for occluded coronary arteries, *NA* not applicable, *TIMI* thrombolysis in myocardial infarction.

^a^PLATO-defined bleeding followed the definitions of bleedings used in the PLATO trial^8^.

**Figure 2:**
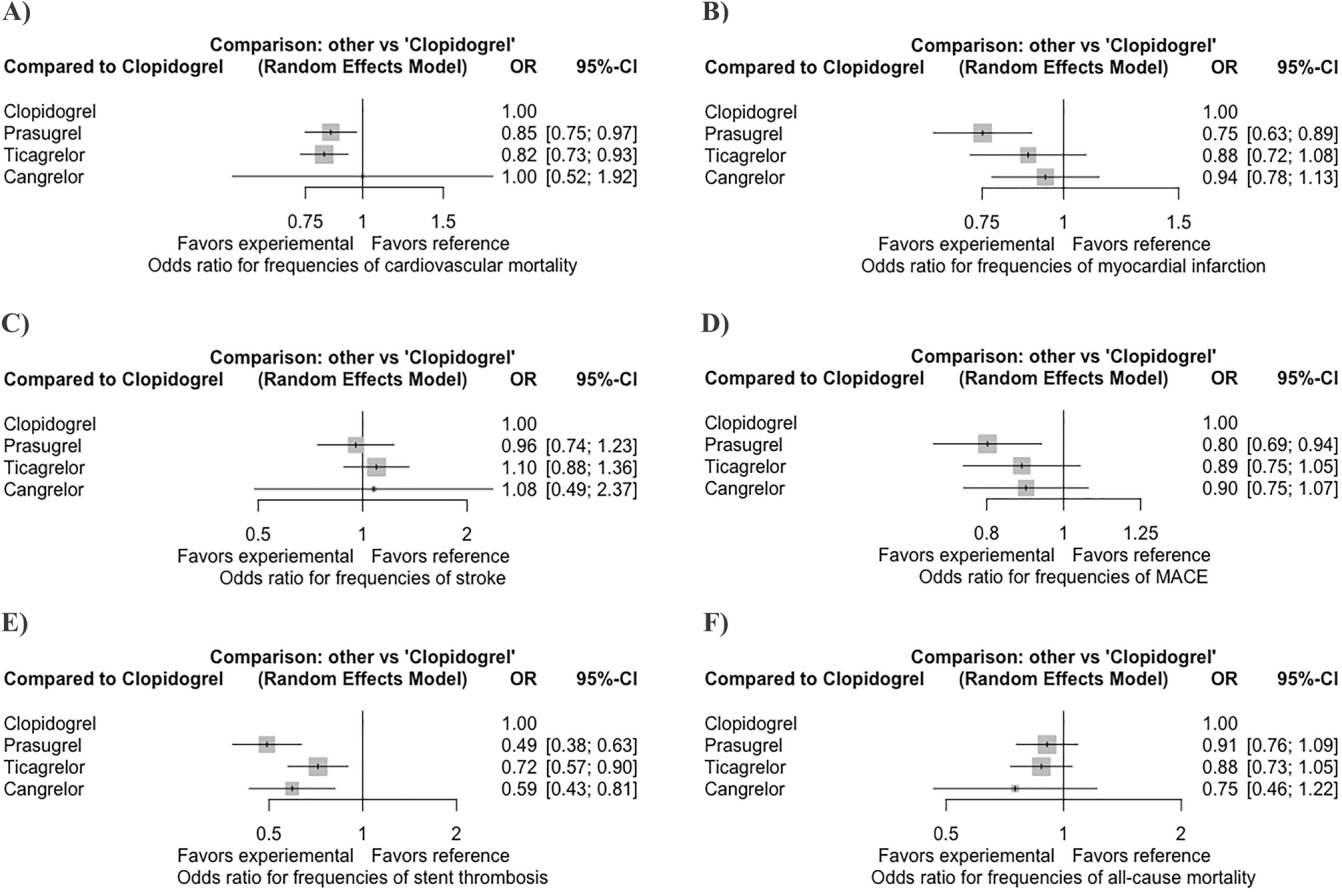
Forest plots assessing the effects of different P2Y_12_ inhibitors relative to clopidogrel. (**A**) Cardiovascular mortality. (**B**) Myocardial infarction. (**C**) Stroke. (**D**) MACE. (**E**) Definite or probable stent thrombosis. (**F**) All-cause mortality. MACE = major adverse cardiovascular events. Square markers indicate odds ratios for cardiovascular outcomes comparing different P2Y_12_ inhibitors to clopidogrel. The horizontal lines indicate 95% confidence intervals.

Our results showed that when compared with clopidogrel, cardiovascular mortality was reduced with both prasugrel (p = 0.015) and ticagrelor (p = 0.002) (Fig. 2A). MI (p = 0.001) and MACE (p = 0.006) were reduced by prasugrel only (Fig. 2B,D). There were fewer definite or probable stent thromboses with prasugrel (p < 0.001), ticagrelor (p = 0.003), and cangrelor (p = 0.001), respectively (Fig. 2E). In addition, there were significantly fewer definite or probable stent thromboses with prasugrel than ticagrelor (p = 0.014) (Table 2). No significant difference was found between clopidogrel, ticagrelor, prasugrel, and cangrelor with respect to the risk of stroke and all-cause death (Table 2, Fig. 2C,F).

**Table 2:** Effect of P2Y_12_ inhibitors on frequencies of clinical outcomes in ACS patients.

	Clopidogrel	Prasugrel	Ticagrelor	Cangrelor
**Myocardial infarction**				
Clopidogrel	1.00	**0.75 (0.63–0.89)**	0.88 (0.72–1.08)	0.94 (0.78–1.13)
Prasugrel	**1.33 (1.12–1.58)**	1.00	1.17 (0.93–1.48)	1.25 (0.97–1.61)
Ticagrelor	1.13 (0.92–1.39)	0.85 (0.67–1.07)	1.00	1.06 (0.80–1.40)
Cangrelor	1.07 (0.88–1.29)	0.80 (0.62–1.04)	0.94 (0.71–1.24)	1.00
**Stroke**				
Clopidogrel	1.00	0.96 (0.74–1.23)	1.10 (0.88–1.36)	1.08 (0.49–2.37)
Prasugrel	1.05 (0.81–1.35)	1.00	1.15 (0.85–1.55)	1.13 (0.49–2.59)
Ticagrelor	0.91(0.74–1.13)	0.87 (0.64–1.18)	1.00	0.98 (0.43–2.23)
Cangrelor	0.93 (0.42–2.05)	0.89 (0.39–2.04)	1.02 (0.45–2.31)	1.00
**Cardiovascular mortality**				
Clopidogrel	1.00	**0.85 (0.75–0.97)**	**0.82 (0.73–0.93)**	1.00 (0.52–1.92)
Prasugrel	**1.17 (1.03–1.34)**	1.00	0.97 (0.83–1.13)	1.17 (0.60–2.29)
Ticagrelor	**1.21 (1.08–1.37)**	1.03 (0.89–1.21)	1.00	1.21 (0.62–2.36)
Cangrelor	1.00 (0.52–1.92)	0.85 (0.44–1.66)	0.82 (0.42–1.60)	1.00
**MACE**				
Clopidogrel	1.00	**0.80 (0.69–0.94)**	0.89 (0.75–1.05)	0.90 (0.75–1.07)
Prasugrel	**1.25 (1.07–1.46)**	1.00	1.10 (0.91–1.34)	1.12 (0.88–1.42)
Ticagrelor	1.13 (0.95–1.34)	0.91 (0.75–1.10)	1.00	1.01 (0.79–1.30)
Cangrelor	1.12 (0.93–1.34)	0.89 (0.70–1.14)	0.99 (0.77–1.27)	1.00
**Definite or probable stent thrombosis**				
Clopidogrel	1.00	**0.49 (0.38–0.63)**	**0.72 (0.57–0.90)**	**0.59 (0.43–0.81)**
Prasugrel	**2.03 (1.58–2.62)**	1.00	**1.46 (1.08–1.97)**	1.20 (0.80–1.81)
Ticagrelor	**1.39 (1.12–1.74)**	**0.69 (0.51–0.93)**	1.00	0.83 (0.56–1.22)
Cangrelor	**1.69 (1.23–2.32)**	0.83 (0.55–1.25)	1.21 (0.82–1.78)	1.00
**All-cause mortality**				
Clopidogrel	1.00	0.91 (0.76–1.09)	0.88 (0.73–1.05)	0.75 (0.46–1.22)
Prasugrel	1.10 (0.92–1.32)	1.00	0.97 (0.78–1.20)	0.83 (0.50–1.38)
Ticagrelor	1.14 (0.95–1.37)	1.03 (0.83–1.28)	1.00	0.86 (0.51–1.43)
Cangrelor	1.33 (0.82–2.15)	1.21 (0.72–2.02)	1.17 (0.70–1.95)	1.00

Results are the Odds Ratios (95% Confidence Interval) in the column-defining therapy compared with the Odds Ratios in the row-defining therapy. For efficacy and safety, Odds Ratio < 1 favors the column-defining therapy. Significant results are shown in bold.

*ACS* acute coronary syndrome, *MACE* major cardiovascular events.

In contrast, there were more all major bleeds with prasugrel (p = 0.012) when compared to clopidogrel. The risk of TIMI major bleeding was significantly increased with prasugrel when compared to clopidogrel (p = 0.003) and ticagrelor (p = 0.014). Compared to clopidogrel, the risk of TIMI minor bleeding was significantly increased with prasugrel (p < 0.001) and cangrelor (p = 0.046). There were also more TIMI minor bleeds with prasugrel than ticagrelor (p = 0.043). Moreover, an increase in the risk of non-CABG-related TIMI major bleeding was found with prasugrel (p = 0.033) and ticagrelor (p = 0.025) when compared with clopidogrel. The risk of CABG-related bleeding was increased with prasugrel when compared to clopidogrel (p = 0.030) and ticagrelor (p = 0.014) (Table 3).

**Table 3:** Effect of different P2Y_12_ inhibitors on risk of bleeding in ACS patients established by network meta-analysis using random-effects models.

	Clopidogrel	Prasugrel	Ticagrelor	Cangrelor
**All major bleeding**				
Clopidogrel	1.00	**1.24 (1.05–1.48)**	1.07 (0.97–1.19)	1.01 (0.59–1.74)
Prasugrel	**0.80 (0.68–0.95)**	1.00	0.86 (0.72–1.03)	0.81 (0.46–1.43)
Ticagrelor	0.93 (0.84–1.03)	1.16 (0.97–1.39)	1.00	0.94 (0.55–1.63)
Cangrelor	0.99 (0.58–1.69)	1.23 (0.70–2.16)	1.06 (0.61–1.83)	1.00
**TIMI major bleeding**				
Clopidogrel	1.00	**1.36 (1.11–1.66)**	1.02 (0.92–1.14)	1.01 (0.59–1.74)
Prasugrel	**0.73 (0.60–0.90)**	1.00	**0.75 (0.60–0.95)**	0.75 (0.42–1.32)
Ticagrelor	0.98 (0.88–1.09)	**1.33 (1.06–1.67)**	1.00	0.99 (0.57–1.71)
Cangrelor	0.99 (0.58–1.69)	1.34 (0.76–2.38)	1.01 (0.58–1.75)	1.00
**Non-CABG-related TIMI major bleeding**				
Clopidogrel	1.00	**1.26 (1.02–1.55)**	**1.25 (1.01–1.52)**	1.00 (0.29–3.45)
Prasugrel	**0.80 (0.64–0.98)**	1.00	0.99 (0.75–1.32)	0.80 (0.23–2.80)
Ticagrelor	**0.80 (0.66–0.97)**	1.01 (0.76–1.34)	1.00	0.80 (0.23–2.81)
Cangrelor	1.00 (0.29–3.46)	1.26 (0.36–4.42)	1.25 (0.36–4.38)	1.00
**CABG-related TIMI major bleeding**				
Clopidogrel	1.00	**1.32 (1.03–1.69)**	0.93 (0.81–1.06)	NA^a^
Prasugrel	**0.76 (0.59–0.97)**	1.00	**0.70 (0.53–0.93)**	NA^a^
Ticagrelor	1.08 (0.95–1.23)	**1.42 (1.07–1.89)**	1.00	NA^a^
Cangrelor	NA^a^	NA^a^	NA^a^	1.00
**TIMI minor bleeding**				
Clopidogrel	1.00	**1.44 (1.16–1.77)**	1.09 (0.93–1.28)	**1.47 (1.01–2.16)**
Prasugrel	**0.70 (0.56–0.86)**	1.00	**0.76 (0.58–0.99)**	1.03 (0.66–1.59)
Ticagrelor	0.92 (0.78–1.08)	**1.32 (1.01–1.72)**	1.00	1.35 (0.89–2.04)
Cangrelor	**0.68 (0.46–0.99)**	0.97 (0.63–1.50)	0.74 (0.49–1.12)	1.00

Results are the Odds Ratios (95% Confidence Interval) in the column-defining therapy compared with the Odds Ratios in the row-defining therapy. For efficacy and safety, Odds Ratio <1 favors the column-defining therapy. Significant results are shown in bold.

*ACS* acute coronary syndrome, *CABG* coronary artery bypass grafting, *NA* not applicable, *TIMI* thrombolysis in myocardial infarction.

^a^CABG-related TIMI major bleeding was not reported in any comparison involving cangrelor.

P-score ranked prasugrel having the highest likelihood for reducing MI (95.5%), stroke (68.7%), MACE (88.6%), and definite or probable stent thrombosis (99.6%). Ticagrelor had the highest rank probabilities for protecting against cardiovascular death (79.3%) while cangrelor had the highest rank probabilities for protecting against all-cause death (78.8%). Although clopidogrel had the lowest likelihood in reducing all the cardiovascular outcomes above, it was ranked the best in reducing all major bleeding (80.9%), TIMI major bleeding (72.8%) and TIMI minor bleeding (94.4%) among all the P2Y_12_ inhibitors assessed (Supplementary Table S5 online).

There was not a high degree of heterogeneity among studies (Supplementary Table S6 online). Significant heterogeneity in the pairwise meta-analysis was found for MACE (I^2^ = 53%, p = 0.0001) in the comparison between ticagrelor vs. clopidogrel, which was due to the PHILO trial^24^. Excluding it could reduce I^2^ to 39% (p = 0.15) (Supplementary Table S7 online). There was also significant heterogeneity in the comparison between cangrelor vs. clopidogrel for MACE (I^2^ = 65%, p = 0.06) and MI (I^2^ = 67%, p = 0.05), driven by the Cangrelor versus Standard Therapy to Achieve Optimal Management of Platelet Inhibition (CHAMPION) PCI trial^10^. I^2^ could be reduced to 0% after excluding it (p = 0.48 and p = 0.36, respectively) (Supplementary Table S8 and Table S9 online). There was no significant inconsistency between direct and indirect comparisons in any of the outcomes assessed (Supplementary Tables S10–S17 online). Using fixed-effects instead of random-effects models, or Bayesian instead of frequentist analysis showed similar results (Supplementary Table S18 and Table S19 online). Inspection of the funnel plots did not reveal any significant publication bias or small study effects (Supplementary Figs. S2–S10 and Supplementary Table S20 online).

Subgroup analysis of oral P2Y_12_ inhibitors and intravenous cangrelor showed consistent results with our main analysis. Interestingly, the effect of ticagrelor on reducing all-cause mortality became significant (OR 0.86, 95% CI 0.74–0.99, p = 0.041) when compared to clopidogrel (Supplementary Table S21 and Supplementary Fig. S11 online).

## Discussion

Our network meta-analysis included the most recent RCTs to compare the efficacy and safety of newer P2Y_12_ inhibitors with clopidogrel in ACS patients, especially aiming to compare different newer P2Y_12_ inhibitors through both direct and indirect evidence. There were three main findings that provided new and further evidence on the efficacy and safety profiles of newer P2Y_12_ inhibitors. First, prasugrel was more beneficial in reducing MACE, MI and definite or probable stent thrombosis but resulted in a significantly higher risk of major and minor bleeding. Secondly, ticagrelor reduced definite or probable stent thrombosis and cardiovascular mortality without increasing the risk of bleeding. Finally, cangrelor reduced definite or probable stent thrombosis without additional cardiovascular benefits but caused more TIMI minor bleeds than clopidogrel.

Our findings are consistent with previous network meta-analyses^35–39^ and meta-analyses^40–42^. Newer P2Y_12_ inhibitors are significantly more effective than clopidogrel in reducing cardiovascular events and cardiovascular deaths in patients with ACS or undergoing PCI. However, previous studies neither compare individual P2Y_12_ inhibitors simultaneously nor include cangrelor for analysis. Our network meta-analysis, which includes the latest evidence, in particular, the two direct trials comparing prasugrel and ticagrelor, provides an important update on the efficacy and safety profiles of P2Y_12_ inhibitors that are already widely used.

Direct evidence showed that prasugrel numerically reduced cardiovascular events when compared to clopidogrel. The biggest trial comparing prasugrel and clopidogrel, the Trial to Assess Improvement in Therapeutic Outcomes by Optimizing Platelet Inhibition with Prasugrel–Thrombolysis in Myocardial Infarction (TRITON-TIMI) 38^6^, however, was the only trial that reported significant reduction of MACE, MI and stent thrombosis with prasugrel. These benefits were confirmed in our network meta-analysis with greater statistical power.

Whether prasugrel increases bleeding events and whether this harmful effect counterbalances its cardiovascular benefits is controversial. The significantly increased risk of non-CABG-related TIMI major bleeding (p = 0.03) with prasugrel, including life-threatening bleeding (p = 0.01) and fatal bleeding (p = 0.002) was observed in ACS patients in TRITON-TIMI 38^6^ but not in the other three prasugrel vs. clopidogrel trials included in this network meta-analysis^29–31^. Besides, the significant excess in non-CABG-related TIMI bleeding was not seen in STEMI patients undergoing PCI^43,44^. Contradictory findings in some observational studies reported the lower incidence of major bleeding with prasugrel^45,46^. Our network meta-analysis provided a more reliable conclusion by integrating direct and indirect evidence for analysis. The cardiovascular benefits of prasugrel were found at the expense of excessive all major bleeding and TIMI bleeding including both CABG-related and non-CABG-related TIMI major bleeding and TIMI minor bleeding. The use of prasugrel should therefore be avoided in patients at high risk of bleeding, including those with prior stroke or transient ischemic attack, those undergoing CABG or other surgeries, those with trauma or at risk from falls, those with cancer, those age 75 or older, or those who weigh less than 60 kg^15^.

PLATO, the biggest trial comparing ticagrelor and clopidogrel, reported a significant reduction in MACE (p < 0.001) and all-cause mortality (p = 0.010) with ticagrelor^8^. These benefits, however, were neither seen in the other four out of seven ticagrelor vs. clopidogrel trials included^23,24,26,28^ nor in this network meta-analysis. Subgroup analysis of oral P2Y_12_ inhibitors only suggested a lower risk of all-cause mortality but not MACE associated with ticagrelor use. The possible explanations for these inconsistent results could be due to the small sample size and the imbalance in clinical characteristics of patients, and the low number of events observed due to the short follow-up period in those trials, unlike PLATO. Our network meta-analysis found a significantly lower risk of stent thrombosis and cardiovascular death with ticagrelor. However, no significant increase in the risk of bleeding events was found, which was consistent across all the included ticagrelor vs. clopidogrel trials, supporting ticagrelor as an effective and safe antiplatelet therapy.

POPular AGE, the first RCT comparing clopidogrel vs. ticagrelor or prasugrel in NSTE-ACS patients aged 70 years and over has just released its results^47^. Clopidogrel was found to be better than ticagrelor for the elderly due to fewer bleeding events. Although this trial treated the 5% of the trial patients prescribed prasugrel as the ticagrelor group, including this trial for analysis did not alter our conclusions (Supplementary Table S22 online). However, this study emphasized the importance of bleeding considerations in the elderly. Clopidogrel could be an alternative strategy for those patients if bleeding risk is extremely high.

A standard-dose of ticagrelor, namely, 180 mg loading dose followed by 90 mg twice daily was used in the ticagrelor trials included for analysis. The PEGASUS-TIMI 54 trial^15^ evaluating two maintaining doses, namely 90 mg twice daily vs. 60 mg twice daily of ticagrelor therapy suggested an improved efficacy but a similar degree of safety (bleeding) with a low-dose ticagrelor, which therefore, might offer a more attractive cost-effective option for patients.

There have been few trials comparing prasugrel and ticagrelor. The Prasugrel versus Ticagrelor in Patients with Acute Myocardial Infarction Treated with Primary Percutaneous Coronary Intervention (PRAGUE)-18^18^ and the Intracoronary Stenting and Antithrombotic Regimen: Rapid Early Action for Coronary Treatment (ISAR-REACT) 5^19^ are the only two RCTs available, but they showed inconsistent findings. PRAGUE-18 reported similar safety and efficacy between prasugrel and ticagrelor in patients with STEMI. In contrast, ISAR-REACT 5 found that the incidence of death, MI, or stroke was significantly lower with prasugrel than ticagrelor, although the incidence of major bleeding was not significantly different across the whole spectrum of presentation of ACS patients. PRAGUE-18 was criticized for the premature termination and the high incidence of switching treatment drugs to clopidogrel after discharge, resulting in the unreliable comparison of clinical outcomes with prasugrel and ticagrelor during the 1-year follow-up period. In addition, two trials lacked adequate statistical power and did not allow the conduction of pairwise meta-analysis. This network meta-analysis took advantage of the indirect evidence from the comparison between these two P2Y_12_ inhibitors with clopidogrel. Our findings showed similar benefits of ticagrelor and prasugrel in reducing the risk of cardiovascular death while prasugrel was better than ticagrelor in reducing the risk of all the cardiovascular events. However, ticagrelor was associated with significantly fewer TIMI major and TIMI minor bleeds than prasugrel. The greater ischemic benefits with prasugrel were offset by the excessive bleeding events. These findings further favoring ticagrelor with a better safety profile among the newer P2Y_12_ inhibitors supported the recommendations in the current guidelines for using ticagrelor as the preferred treatment over the other P2Y_12_ inhibitors for ACS patients regardless of the initial treatment strategy^16,17^. The upcoming trials in the future will provide more evidence assessing the comparative effects between ticagrelor and prasugrel.

Unlike prasugrel and ticagrelor, cangrelor is not an oral drug and only used in a peri-procedural setting. It requires the administration of oral P2Y_12_ inhibitors, usually clopidogrel, after discontinuation of the cangrelor infusion. The cardiovascular effects of cangrelor were inconsistent in the CHAMPION studies^10,11,13^. Few clinical trials compared cangrelor with ticagrelor and prasugrel. In this network meta-analysis maximizing the use of direct and indirect evidence, cangrelor was found to reduce stent thrombosis but increase TIMI minor bleeding. Previous patient-level meta-analysis integrating these three studies agreed with our findings. It reported a 41% (p < 0.001) reduction in the odds of stent thrombosis and a 51% (p = 0.022) increase in the odds of TIMI minor bleeding with cangrelor, irrespective of the patient clinical presentations^48^. CHAMPION PCI^10^ and CHAMPION PLATFORM^11^ were given more weight to the unfavorable results of cangrelor. The definition of periprocedural MI used in these two trials did not allow for discrimination of re-infarction in patients that presented for PCI shortly after admission with a biomarker-positive ACS, leading to a decrease in the number of trial events^49^. Moreover, the increased TIMI minor bleeding found in our analysis but not in the CHAMPION studies could be driven by the indirect evidence and due to the increased statistical power. Although cangrelor has not been adequately studied in head-to-head comparisons with prasugrel and ticagrelor, no superiority of cangrelor over these two drugs has been identified by using indirect evidence for analysis in this network, which needs to be further confirmed in large direct comparative trials in the future. The increased speed of onset and the rapid reversibility of cangrelor offer an alternative to loading with oral P2Y_12_ inhibitors in the acute phase of PCI. Moreover, unlike oral P2Y_12_ inhibitors which need a wash-out period of 5–7 days^1^, cangrelor is an attractive option for those patients awaiting surgery.

Intensive inhibition of platelet aggregation is usually accompanied by an increase in bleeding. Newer P2Y_12_ inhibitors that result in more intensive inhibition of platelet aggregation are more likely to have a higher rate of bleeding than clopidogrel. The use of newer P2Y_12_ inhibitors, especially prasugrel, requires a tradeoff between decreasing ischemic risks with an increased bleeding risk for each individual patient^50^. In patients who are not at risk of bleeding or can tolerate therapy without increased bleeding events, and in patients for whom reducing the high risk of ischemic events outweighs the excess bleeding, prasugrel can be considered. Ticagrelor, with its better safety profile, should be considered over clopidogrel in most ACS patients. Although, current observational studies reporting inconsistent results do not confirm the superiority of newer P2Y_12_ inhibitors over clopidogrel in ACS patients receiving PCI^51^, they are prone to bias and should be interpreted with caution given the potential for confounding. Nevertheless, real-world studies are helpful in translating the findings from RCTs into practice settings in the general population.

A limitation of this network meta-analysis is the lack of patient-level data, so it was unable to adjust the analysis for the severity of presentation, the cardiovascular risk profile of patients, revascularization strategy, and use of other medications. Analysis dedicated to specific patient subgroups could help delineate appropriate therapies in different populations. Moreover, the comparative results among newer P2Y_12_ inhibitors were mostly driven by indirect evidence due to the scarcity of head-to-head comparative trials. Inevitably, trials included in the network meta-analysis varied in patient characteristics, comorbidities, treatment regimens, follow-up periods, and definitions of outcomes.

## Conclusions

Prasugrel and ticagrelor both show greater potentials than clopidogrel in protecting against stent thrombosis and cardiovascular death in ACS patients. Prasugrel is more beneficial in reducing MACE, MI, especially in reducing definite or probable stent thrombosis among the oral P2Y_12_ inhibitors but at the expense of increased major and minor bleeding. Intravenous cangrelor lowers the risk of definite or probable stent thrombosis compared with clopidogrel whereas increases TIMI minor bleeding. The treatment of ACS patients should take the benefit-risk profile of each individual patient into account. Ticagrelor not increasing bleeding is therefore the antiplatelet drug of choice for most ACS patients. Prasugrel can be recommended to those ACS patients at high ischemic risk but low bleeding risk.

## Supplementary information

Supplementary Information.
