# Supplementary material for: Efficacy and safety of newer P2Y12 inhibitors for acute coronary syndrome: a network meta-analysis
Source: Sci Rep. 2020 Oct 8;10:16794. doi: 10.1038/s41598-020-73871-x (PMC7545197; doi:10.1038/s41598-020-73871-x)
Supplement: Supplementary file 1 — Supplementary Information. [file 41598_2020_73871_MOESM1_ESM.pdf]

Supplementary Information

Title: Efficacy and Safety of Newer $P2Y_{12}$ Inhibitors for Acute Coronary Syndrome: A Network Meta-analysis

Author list: Yue FEI, PhD[1]; Cheuk Kiu LAM, MBBS[1]; Bernard Man Yung CHEUNG, PhD[1,2,3,*]

[1] Division of Clinical Pharmacology and Therapeutics, Department of Medicine, The University of Hong Kong, Pokfulam, Hong Kong, China.

[2] State Key Laboratory of Pharmaceutical Biotechnology, The University of Hong Kong, Pokfulam, Hong Kong, China.

3 Institute of Cardiovascular Science and Medicine, The University of Hong Kong, Pokfulam, Hong Kong, China.

Address for correspondence:

Prof Bernard M Y Cheung

Email: mycheung@hku.hk

University Department of Medicine, Queen Mary Hospital

102 Pokfulam Road, Hong Kong, China

Tel: +85222554347

Fax: +8522818647

Table of Content

TABLE OF CONTENT 2

SUPPLEMENTARY TABLE S1. SUMMARY FOR BLINDING, RANDOMISATION AND PLACEBO CONTROL OF STUDIES 6

SUPPLEMENTARY TABLE S2. RISK OF BIAS REPORTING 8

SUPPLEMENTARY TABLE S3. PATIENTS' BASELINE CHARACTERISTICS 9

SUPPLEMENTARY TABLE S4. DEFINITION OF MAJOR ADVERSE CARDIOVASCULAR EVENTS IN EACH TRIAL INCLUDED 12

SUPPLEMENTARY TABLE S5. P-SCORE INDICATING THE PROBABILITY THAT THE $P2Y_{12}$ INHIBITOR IS THE BEST FOR THE OUTCOME 16

SUPPLEMENTARY TABLE S6. ESTIMATES OF BETWEEN TRIAL HETEROGENEITY 17

SUPPLEMENTARY TABLE S7. SENSITIVITY ANALYSIS OF THE EFFECT OF $P2Y_{12}$ INHIBITORS ON FREQUENCY OF MAJOR CARDIOVASCULAR EVENTS (MACE) IN THE COMPARISON BETWEEN TICAGRELOR VS. CLOPIDOGREL 18

SUPPLEMENTARY TABLE S8. SENSITIVITY ANALYSIS OF THE EFFECT OF $P2Y_{12}$ INHIBITORS ON FREQUENCY OF MAJOR CARDIOVASCULAR EVENTS (MACE) IN THE COMPARISON BETWEEN CANGRELOR VS. CLOPIDOGREL 19

SUPPLEMENTARY TABLE S9. SENSITIVITY ANALYSIS OF THE EFFECT OF $P2Y_{12}$ INHIBITORS ON FREQUENCY OF MYOCARDIAL INFARCTION IN THE COMPARISON BETWEEN CANGRELOR VS. CLOPIDOGREL 20

SUPPLEMENTARY TABLE S10. NODE-SPLITTING RESULTS OF TESTING CONSISTENCY FOR MAJOR ADVERSE CARDIOVASCULAR EVENTS (MACE) USING RANDOM-EFFECTS MODEL 21

SUPPLEMENTARY TABLE S11. NODE-SPLITTING RESULTS OF TESTING CONSISTENCY FOR MYOCARDIAL INFARCTION USING RANDOM-EFFECTS MODEL 22

SUPPLEMENTARY TABLE S12. NODE-SPLITTING RESULTS OF TESTING CONSISTENCY FOR STROKE USING RANDOM-EFFECTS MODEL 23

SUPPLEMENTARY TABLE S13. NODE-SPLITTING RESULTS OF TESTING CONSISTENCY FOR CARDIOVASCULAR MORTALITY USING RANDOM-EFFECTS MODEL 24

SUPPLEMENTARY TABLE S14. NODE-SPLITTING RESULTS OF TESTING CONSISTENCY FOR DEFINITE OR PROBABLE STENT THROMBOSIS USING RANDOM-EFFECTS MODEL 25

SUPPLEMENTARY TABLE S15. NODE-SPLITTING RESULTS OF TESTING CONSISTENCY FOR THROMBOLYSIS IN MYOCARDIAL INFARCTION (TIMI) MAJOR BLEEDING USING RANDOM-EFFECTS MODEL 26

SUPPLEMENTARY TABLE S16. NODE-SPLITTING RESULTS OF TESTING CONSISTENCY FOR THROMBOLYSIS IN MYOCARDIAL INFARCTION (TIMI) MINOR BLEEDING USING RANDOM-EFFECTS MODEL 27

SUPPLEMENTARY TABLE S17. NODE-SPLITTING RESULTS OF TESTING CONSISTENCY FOR ALL-CAUSE MORTALITY USING RANDOM-EFFECTS MODEL 28

SUPPLEMENTARY TABLE S18. EFFECT OF DIFFERENT $P2Y_{12}$ INHIBITORS ON FREQUENCIES OF ALL OUTCOMES IN ACS PATIENTS ESTABLISHED BY NETWORK META-ANALYSIS USING FIXED-EFFECTS MODELS 29

SUPPLEMENTARY TABLE S19. ESTIMATES OF RISK OF OUTCOMES BETWEEN DIFFERENT $P2Y_{12}$ INHIBITORS USING A BAYESIAN FRAMEWORK

WITH NON-INFORMATIVE PRIORS 32

SUPPLEMENTARY TABLE S20. ANALYSIS OF FUNNEL PLOTS IN THROMBOLYSIS IN SUPPLEMENTARY FIGS. S2-S9 TO ASSESS PUBLICATION BIAS 34

SUPPLEMENTARY TABLE S21. EFFECT OF ORAL $P2Y_{12}$ INHIBITORS ON FREQUENCIES OF CLINICAL OUTCOMES IN ACS PATIENTS ESTABLISHED BY NETWORK META-ANALYSIS 36

SUPPLEMENTARY TABLE S22. EFFECT OF $P2Y_{12}$ INHIBITORS ON FREQUENCIES OF CLINICAL OUTCOMES IN ACS PATIENTS INCLUDING THE POPULAR AGE TRIAL 37

SUPPLEMENTARY FIG. S1. FLOW DIAGRAM OF SCIENTIFIC LITERATURE SEARCH AND STUDY SELECTION. 39

SUPPLEMENTARY FIG. S2. FUNNEL PLOT SHOWING PUBLICATION BIAS FOR THE EFFECT OF DIFFERENT $P2Y_{12}$ INHIBITORS ON FREQUENCIES OF MAJOR ADVERSE CARDIOVASCULAR EVENTS (MACE) IN PATIENTS WITH ACUTE CORONARY SYNDROME. 40

SUPPLEMENTARY FIG. S3. FUNNEL PLOT SHOWING PUBLICATION BIAS FOR THE EFFECT OF DIFFERENT $P2Y_{12}$ INHIBITORS ON FREQUENCIES OF CARDIOVASCULAR MORTALITY IN PATIENTS WITH ACUTE CORONARY SYNDROME 41

SUPPLEMENTARY FIG. S4. FUNNEL PLOT SHOWING PUBLICATION BIAS FOR THE EFFECT OF DIFFERENT $P2Y_{12}$ INHIBITORS ON FREQUENCIES OF MYOCARDIAL INFARCTION IN PATIENTS WITH ACUTE CORONARY SYNDROME 42

SUPPLEMENTARY FIG S5. FUNNEL PLOT SHOWING PUBLICATION BIAS FOR THE EFFECT OF DIFFERENT $P2Y_{12}$ INHIBITORS ON FREQUENCIES OF DEFINITE AND PROBABLE STENT THROMBOSIS IN PATIENTS WITH ACUTE CORONARY SYNDROME 43

SUPPLEMENTARY FIG. S6. FUNNEL PLOT SHOWING PUBLICATION BIAS FOR THE EFFECT OF DIFFERENT $P2Y_{12}$ INHIBITORS ON FREQUENCIES OF ALL-CAUSE MORTALITY IN PATIENTS WITH ACUTE CORONARY SYNDROME 44

SUPPLEMENTARY FIG. S7. FUNNEL PLOT SHOWING PUBLICATION BIAS FOR THE EFFECT OF DIFFERENT $P2Y_{12}$ INHIBITORS ON FREQUENCIES OF STROKE IN PATIENTS WITH ACUTE CORONARY SYNDROME 45

SUPPLEMENTARY FIG. S8. FUNNEL PLOT SHOWING PUBLICATION BIAS FOR THE EFFECT OF DIFFERENT $P2Y_{12}$ INHIBITORS ON FREQUENCIES OF THROMBOLYSIS IN MYOCARDIAL INFARCTION (TIMI) MAJOR BLEEDING IN PATIENTS WITH ACUTE CORONARY SYNDROME 46

SUPPLEMENTARY FIG. S9. FUNNEL PLOT SHOWING PUBLICATION BIAS FOR THE EFFECT OF DIFFERENT $P2Y_{12}$ INHIBITORS ON FREQUENCIES OF THROMBOLYSIS IN MYOCARDIAL INFARCTION (TIMI) MINOR BLEEDING IN PATIENTS WITH ACUTE CORONARY SYNDROME........ 47

SUPPLEMENTARY FIG. S10. FUNNEL PLOT SHOWING PUBLICATION BIAS FOR THE EFFECT OF DIFFERENT $P2Y_{12}$ INHIBITORS ON FREQUENCIES OF ALL MAJOR BLEEDING IN PATIENTS WITH ACUTE CORONARY SYNDROME........ 48

SUPPLEMENTARY FIG. S11. FOREST PLOTS OF THE EFFECTS OF CANGRELOR ON A). MACE; B). MYOCARDIAL INFARCTION; C). DEFINITE OR PROBABLE STENT THROMBOSIS; D). ALL-CAUSE MORTALITY; E). TIMI MAJOR BLEEDING; F). TIMI MINOR BLEEDING. 49

Supplementary Table S1. Summary for blinding, randomisation and placebo control of studies

Study	Blinding	Randomisation assignment	Presence of Placebo	Time of randomisation
DISPERSE-2	Double-blinded	Not reported	With placebo control	Not reported
PLATO	Double-blinded	Patients were randomly assigned, using an interactive voice response system and a blocking size of four, to ticagrelor or clopidogrel in a one-to-one ratio in a double-blind, double-dummy design. The randomisation schedule was created by the AstraZeneca GRAND system.	With placebo control	Not reported
PHILO	Double-blinded	Randomisation was performed using a central interactive web response or voice system.	With placebo control	Not reported
Tang et al	Double-blinded	Randomisation was performed using envelope method.	No placebo control	Not reported
SETFAST	Open-labelled	Not reported	No placebo control	At the time of diagnostic angiogram
Wang et al	Double-blinded	Not reported	No placebo control	With the least delay after admission
TREAT	Open-labelled	Randomisation was performed in a concealed fashion with the use of an automated web-based system, in permuted blocks of 4, stratified according to site.	No placebo control	After the index event and <24 hrs after the event
JUMBO–TIMI 26	Double-blinded	Not reported	No placebo control	After diagnostic catheterization
TRILOGY ACS	Double-blinded	Interactive voice- response system	No placebo control	Not reported
TRITON-TIMI 38	Double-blinded	Patients were randomly allocated by interactive voice response system (initiated by research nurse or study personnel at every local site) to either prasugrel or clopidogrel.	No placebo control	Not reported

PRASFIT-ACS	Double-blinded	Randomisation was performed using a uniform random numbers list generated using SAS software (SAS Institute, Cary, NC, USA).	No placebo control	After patients provided written informed consent
CHAMPION PCI	Double-blinded	Patients were randomly assigned to either cangrelor or clopidogrel in a 1:1 double-blind, double-dummy design with the use of an interactive voice-response system.	With placebo control	Not reported
CHAMPION PLATFORM	Double-blinded	Not reported	With placebo control	Not reported
CHAMPION PHOENIX	Double-blinded	Randomisation was performed with the use of an interactive voice-response or Web-response system, with stratification according to site, baseline status (normal or abnormal, as defined by a combination of bio-marker levels, electrocardiographic changes, and symptoms), and intended loading dose of clopidogrel (600 mg or 300 mg).	With placebo control	Not reported
PRAGUE-18	Open-labelled	Simple randomisation with GraphPad scientific software was adopted for the study. The sealed envelope method was used for distribution of randomisation codes.	No placebo control	Not reported
ISAR-REACT 5	Open-labelled	In each participating centre, treatment assignments were made with the use of sealed, opaque envelopes containing a computer-generated sequence that had been created at the coordinating centre.	No placebo control	After enrolment

Supplementary Table S2. Risk of Bias Reporting

Study	Sequence generation	Allocation concealment	Blinding of participants and personnel	Blinding of outcome	Incomplete outcome data	Free from other bias
**Prasugrel vs. Clopidogrel**						
TRILOGY ACS	Low	Low	Low	Low	Low	Low
TRITON-TIMI 38	Low	Low	Low	Low	Low	Low
PRASFIT-ACS	Low	Low	Low	Low	Low	Low
JUMBO–TIMI 26	Low	Low	Low	Low	Low	Low
**Ticagrelor vs. Clopidogrel**						
DISPERSE	Low	Low	Low	Low	Low	Low
PLATO	Low	Low	Low	Low	Low	Low
PHILO	Low	Low	Low	Low	Low	Low
Tang et al	Low	Low	Low	Low	Low	Low
SETFAST	Low	Low	Unclear	Unclear	Low	Low
Wang et al	Low	Low	Low	Low	Low	Low
TREAT	Low	Low	Unclear	Low	Low	Low
**Cangrelor vs. Clopidogrel**						
CHAMPION PCI	Low	Low	Low	Low	Low	Low
CHAMPION PLATFORM	Unclear	Low	Low	Low	Low	Low
CHAMPION PHOENIX	Low	Low	Low	Low	Low	Low
**Ticagrelor vs. Prasugrel**						
PRAGUE-18	Low	Low	Unclear	Unclear	Low	Low
ISAR-REACT 5	Low	Low	Low	Low	Low	Low

Each domain of risk was assigned “Low” for low risk, “Unclear” for unclear risk and “High” for high risk.

Supplementary Table S3. Patients' baseline characteristics

Study	$P2Y_{12}$ inhibitors	N	Age (yrs, mean ± SD)	Male (%)	BMI (%)	Race White (%)	Smoker (%)	Loading dose (mg)	Mainte-nance dose (mg)	DM (%)	Prior MI (%)	Prior PCI (%)	Prior CABG (%)	Maxi-mum follow-up
DISPERSE-2	Ticagrelor	334	64.0±12.1	61	28±5.4	95	NR	325	NR	25	24	13	8	12 weeks
	Clopidogrel	327	62.0±11.0	66	29±5.4	94	NR	300	75	25	28	17	11	
PLATO	Ticagrelor	9333	62.0 (Median)	71.6	27 (13-68)*	91.8	36.0	180	90	24.9	20.4	13.6	5.7	12 months
	Clopidogrel	9291	62.0 (Median)	71.7	27 (13-70)*	91.6	35.7	300	75	25.1	20.7	13.1	6.2	
PHILO	Ticagrelor	401	67.0±12.0	76.3	23.7 (15.6-43.4)*	0	37.7	180	90	38.4	8.2	11.2	1.2	12 months
	Clopidogrel	400	66.0±11.0	76.7	23.6 (14.2-38.6)*	0	39.3	300	75	34.1	7.8	10.5	0.3	
Tang et al	Ticagrelor	200	64.3±11.4	71.0	NR	0	58.0	180	90	29.0	8.0	100	0	6 months
	Clopidogrel	200	64.2±11.1	73.0	NR	0	62.0	600	75	21.0	5.0	100	0	
SETFAST	Ticagrelor	76	62.1±10.2	76.3	29.4 ± 5.1	93.4	65.8	180	90	15.8	11.8	10.5	1.3	30 days
	Clopidogrel	68	64.1±14.0	72.1	29.5 ± 5.9	97.1	63.2	300	75	19.1	8.8	2.9	5.9	
Wang et al	Ticagrelor	100	79.0 (76-85)*	69.0	NR	0	37.0	180	90	42.0	17.0	3.0	0	12 months
	Clopidogrel	100	80.0 (74-86)*	66.0	NR	0	41.0	300	75	39.0	15.0	6.0	0	
TREAT	Ticagrelor	1913	59 (51.6-65.2)*	77.4	26.5 (24.0-29.8)*	57.5	NR	180	90	17.4	9.3	5.9	0.8	30 days

	Clopidogrel	1886	58.8 (51.6-65.5)*	76.8	26.5 (24.0-29.4)*	57.11	NR	300-600	75	16.1	8.0	5.3	0.7	
JUMBO–TIMI 26	Prasugrel	650	59	76	29.6	90	23	40 or 60	7.5 or 10 or 15	27	NR	NR	NR	30 days
	Clopidogrel	254	58	76	29.4	94	31	300	75	25	NR	NR	NR	
TRILOGY ACS	Prasugrel	3620	62.0 (56-68)*	63.8	NR	NR	23.3	30	10	38.5	43.3	27.0	14.6	30 months
	Clopidogrel	3623	62.0 (56-68)*	63.2	NR	NR	23.6	300	75	39.3	44.8	29.1	14.3	
TRITON-TIMI 38	Prasugrel	6813	61.0 (53-69)*	75.0	28 (25-31)*	92	38	60	10	23	18	99	1	15 months
	Clopidogrel	6795	61.0 (53-70)*	73.0	28 (25-31)*	93	38	300	75	23	18	99	1	
PRASFIT-ACS	Prasugrel	685	65.4±11.4	78.2	24.2±3.6	NR	39.9	20	3.75	36.5	5.0	95.0	0.9	14 days
	Clopidogrel	678	65.1±11.3	79.4	24.2±3.3	NR	41.2	300	75	35.0	5.2	94.0	0.6	
CHAMPION PCI	Cangrelor	4347	62.0 (54-70)*	73.9	NR	82.7	28.6	30 μg/kg bolus and 4 μg/kg/min infusion	Clopidogrel (600)**	30.5	24.7	28.8	12.6	30 days
	Clopidogrel	4320	62.0 (54-71)[a]	72.3	NR	81.5	29.0	600	Placebo (600)**	30.4	24.7	28.6	12.4	
CHAMPION PLATFORM	Cangrelor	2656	63.0 (54-71)*	72.0	NR	75.7	31.8	30 μg/kg bolus and 4 μg/kg/min infusion	Clopidogrel (600)**	30.8	24.1	14.2	7.5	12 months
	Clopidogrel	2645	63.0 (54-71)*	70.3	NR	75.8	30.4	600	Placebo (600)**	32.6	25.7	15.5	8.4	

CHAMPION PHOENIX	Cangrelor	5470	64.0 (56-72)[*]	71.5	NR	93.8	28.2	30 μg/kg bolus and 4 μg/kg/min infusion	Clopidogrel (600)[**]	27.8	20.1	23.2	10.6	30 days
	Clopidogrel	5469	64.0 (56-72)[*]	72.7	NR	93.7	29.0	600 or 300	Placebo (600)[**]	28.1	21.6	24.4	9.2	
PRAGUE-18	Ticagrelor	596	61.8 (44.6-79.8)[*]	73.7	NR	NR	65.8	180	90	20.8	9.2	7.6	1.5	12 months
	Prasugrel	634	61.8 (42.7-78.7)[*]	77.1	NR	NR	64.0	60	10 or 5 for those >75	20.0	7.4	6.6	1.9	
ISAR-REACT 5	Ticagrelor	2012	64.5±12.0	76.2	27.8±4.6	NR	34.1	180	90	23.0	15.5	22.5	NR	12 months
	Prasugrel	2006	64.6±12.1	76.2	27.8±4.4	NR	33.4	60	10 or 5 for those >75	21.4	16.0	23.1	NR	

Abbreviations used in Supplementary Table S3: BMI = body mass index; CABG = coronary artery bypass grafting; DM = diabetes mellitus; MI = myocardial infarction; NR = not reported; PCI = percutaneous coronary intervention; SD = standard deviation.

[*] Values reported as range.

[**] Cangrelor group: 600-mg clopidogrel capsules were given after the end of 30 μg/kg bolus and 4 μg/kg/min infusion of cangrelor in combination with 600-mg placebo capsules;

Clopidogrel group: 600-mg placebo capsules were given after the end of 30 μg/kg bolus and 4 μg/kg/min infusion of placebo in combination with 600-mg clopidogrel capsules.

Supplementary Table S4. Definition of major adverse cardiovascular events in each trial included

Study	Definition of major adverse cardiovascular events	Definition of myocardial infarction
DISPERSE-2	The composite incidence of myocardial infarction (including silent myocardial infarction), death, stroke, and severe recurrent ischemia	Not applicable
PLATO	The composite of death from vascular causes, myocardial infarction, or stroke	Myocardial infarction was defined in accordance with the universal definition proposed in 2007
PHILO	The composite endpoint of spontaneous myocardial infarction, stroke, or cardiovascular death	Including both periprocedural and spontaneous myocardial infarction; periprocedural myocardial infarction was defined as an elevation of (CK)-myocardial band (MB) ⩾300% that of laboratory specified upper limit of normal (ULN) (with or without development of pathological Q-waves on electrocardiogram), with no symptoms required.
Tang et al	The composite of overall death, myocardial infarction, unplanned revascularization, and stroke	Myocardial infarction was defined according to the Third Universal Definition of Myocardial Infarction
SETFAST	Comprising of death, re-infarction, revascularization, and stroke	Not applicable
Wang et al	The composite of myocardial infarction, stroke, or cardiovascular death	Myocardial infarction was defined in accordance with the Universal Definition proposed in 2007
TREAT	The composite outcome of death from vascular causes, myocardial infarction, or stroke	Recurrent myocardial infarction when cardiac markers were increasing was defined as recurrent cardiac ischemic symptoms and a new ST-elevation.<br>Recurrent myocardial infarction when cardiac markers were stable or decreasing but before cardiac markers have returned to normal was defined as re-elevation of troponin or creatine kinase CK-MB with one of the following criteria:<br>(1). Symptoms of ischemia.<br>(2). New or presumed new significant ST-segment–T wave changes or new left bundle branch block. Development of pathological Q waves in the electrocardiogram.<br>(3). Imaging evidence of new loss of viable myocardium or new regional wall motion abnormality.

		(4). Identification of an intracoronary thrombus by angiography or autopsy.<br>Myocardial infarction after cardiac biomarkers have returned to normal was defined as elevation of biochemical markers above the ULN and with at least one of the above criteria.
JUMBO–TIMI 26	Defined as any one of the following, occurring through the 30-day visit after percutaneous coronary intervention: (1) death (all-cause mortality), (2) myocardial infarction, (3) stroke, (4) recurrent myocardial ischemia requiring hospitalization, and (5) clinical target vessel thrombosis	Myocardial infarction was defined according to the standard American College of Cardiology/American Heart Association definitions.<br>If CK-MB was > the ULN at the time of the suspected event, both an increase by ≥50% over the previous value and documentation that CK-MB was decreasing before the suspected recurrent myocardial infarction were required. Within 24 h after percutaneous coronary intervention (PCI), a subject would be considered to have had a myocardial infarction with the ensuing CK-MB >3 times the ULN; within 24 h of coronary artery bypass grafting (CABG), the threshold was CK-MB >10 times the ULN.<br>Periprocedural myocardial infarction could also be determined by either development of new, abnormal Q waves considered to be distinct from the evolution of an index myocardial infarction or pathological findings of a new myocardial infarction thought to be distinct from a myocardial infarction in evolution before randomisation. If the suspected myocardial infarction was not associated with a procedure, the definition required CK-MB or cardiac troponin greater than ULN and either chest pain or ischemic discomfort lasting >20 min at rest or hemodynamic decompensation.
TRILOGY ACS	The composite of death from cardiovascular causes, nonfatal myocardial infarction, or nonfatal stroke among patients under the age of 75 years	Elevation or re-elevation of the ST-segment; either ischemic symptoms ≥20 min or hemodynamic decompensation Troponin (T or I) or CK-MB > the ULN; either ischemic symptoms ≥20 min or ST-segment deviation ≥1 mm in ≥1 lead myocardial infarction associated with revascularization.<br>New Q waves ≥0.04 s or pathology distinct from the index event.
TRITON-TIMI 38	The composite of cardiovascular death, non-fatal myocardial infarction, or non-fatal stroke	Not applicable
PRASFIT-ACS	The composite of cardiovascular death, nonfatal myocardial infarction, and nonfatal ischemic stroke	Nonfatal myocardial infarction was defined as events fulfilling at least one of the following 3 criteria.

		(1). In patients with normal CK-MB before PCI/CABG, CK-MB had to be (a). ≥3-fold the ULN in 2 samples obtained, or ≥5-fold the ULN in 1 sample obtained <48 h after PCI, or (b). ≥10-fold the ULN in 1 sample obtained <48 h after CABG. Patients whose CK-MB exceeded the ULN before PCI/CABG had to show a transient decrease with a subsequent increase of ≥1.5-fold the previous value and satisfy (a) or (b). (2). More than 48 h after PCI, the CK-MB or troponin levels had to be ≥2-fold the ULN, accompanied by 1 or more of the following: new or recurrent sustained ischemic chest pain, hemodynamic decompensation, or new or recur- rent ST elevation/depression ≥0.1 mV. (3). Abnormal Q waves had to persist for ≥0.04 s.
CHAMPION PCI	The composite of death from any cause, myocardial infarction, or ischemia-driven revascularization	Myocardial infarction was defined by a new Q wave lasting >0.03 s in two contiguous electrocardiographic leads or elevations in CK and the CK-MB fraction, including an increase in the CK-MB level that was ≥3 times the local ULN range and, when biomarkers were elevated before percutaneous coronary intervention, an additional 50% above baseline.
CHAMPION PLATFORM	The composite of death, myocardial infarction, or ischemia-driven revascularization	Myocardial infarction was defined by a new Q wave lasting >0.03 s in two contiguous electrocardiographic leads or elevations in CK and the CK-MB fraction, including an increase in the CK-MB level that was ≥3 times the local ULN range and, when biomarkers were elevated before percutaneous coronary intervention, an additional 50% above baseline.
CHAMPION PHOENIX	The composite rate of death from any cause, myocardial infarction, ischemia-driven revascularization, or stent thrombosis in the 48 hours after randomisation in the modified intention-to-treat population (which comprised patients who actually underwent percutaneous coronary intervention and received the study drug)	Myocardial infarction was defined by a new Q wave lasting >0.03 s in two contiguous electrocardiographic leads or elevations in CK and the CK-MB fraction, including an increase in the CK-MB level that was ≥3 times the local ULN range and, when biomarkers were elevated before percutaneous coronary intervention, an additional 50% above baseline.
PRAGUE-18	The combined occurrence of cardiovascular death, non-fatal myocardial infarction, or stroke	Defined according to the Third Universal Definition of Myocardial Infarction

ISAR-REACT 5	The composite of death, myocardial infarction, or stroke at 1 year after randomisation.	Defined according to the Third Universal Definition of Myocardial Infarction

Supplementary Table S5. P-score indicating the probability that the $P2Y_{12}$ inhibitor is the best for the outcome

	Clopidogrel	Prasugrel	Ticagrelor	Cangrelor
**MACE**	6.67	88.64	53.93	50.77
**Myocardial infarction**	12.25	95.53	54.67	37.54
**Stroke**	57.66	68.71	29.13	44.50
**Cardiovascular mortality**	16.94	66.96	79.31	36.80
**Definite or probable stent thrombosis**	0.08	99.63	39.06	67.23
**All major bleeding**	80.91	9.99	48.31	60.79
**TIMI major bleeding**	72.80	5.53	60.43	61.24
**TIMI minor bleeding**	94.36	18.97	68.23	18.43
**All-cause mortality**	11.49	49.02	60.71	78.79

Abbreviations used in Supplementary Table S5: MACE = major cardiovascular events, TIMI = Thrombolysis In Myocardial Infarction.

The numbers in this table represent the probability (in percent) that each P2Y12 inhibitor is the best for the outcome.

Supplementary Table S6. Estimates of between trial heterogeneity

Outcomes	τ^2
Major adverse cardiovascular events (MACE)	0.0171
Cardiovascular mortality	0
Myocardial infarction	0.0185
Stroke	0
Definite or probable stent thrombosis	0
All major bleeding	0
Thrombolysis in Myocardial Infarction (TIMI) major bleeding	0
TIMI minor bleeding	0
All-cause mortality	0.0124

Supplementary Table S7. Sensitivity analysis of the effect of $P2Y_{12}$ inhibitors on frequency of major cardiovascular events (MACE) in the comparison between ticagrelor vs. clopidogrel

Study	Before excluding study					After excluding study				
	**OR**	**p-value**	**I^2**	**Chi^2**	**P-value**	**OR**	**p-value**	**I^2**	**Chi^2**	**P-value**
DISPERSE-2	0.84 (0.77-0.92)	0.0001	53%	12.65	0.05	0.84 (0.76-0.91)	<0.0001	58%	12.04	0.03
PLATO	0.84 (0.77-0.92)	0.0001	53%	12.65	0.05	0.88 (0.70-1.11)	0.29	59%	12.34	0.03
PHILO	0.84 (0.77-0.92)	0.0001	53%	12.65	0.05	0.83 (0.75-0.90)	<0.0001	39%	8.17	0.15
Tang et al	0.84 (0.77-0.92)	0.0001	53%	12.65	0.05	0.85 (0.77-0.92)	0.0002	46%	9.34	0.10
SETFAST	0.84 (0.77-0.92)	0.0001	53%	12.65	0.05	0.84 (0.77-0.92)	0.0001	55%	11.17	0.05
Wang et al	0.84 (0.77-0.92)	0.0001	53%	12.65	0.05	0.85 (0.77-0.93)	0.0003	50%	9.96	0.08
TREAT	0.84 (0.77-0.92)	0.0001	53%	12.65	0.05	0.83 (0.76-0.91)	0.0001	60%	12.39	0.03

Supplementary Table S8. Sensitivity analysis of the effect of $P2Y_{12}$ inhibitors on frequency of major cardiovascular events (MACE) in the comparison between cangrelor vs. clopidogrel

Study	Before excluding study					After excluding study				
	**OR**	**p-value**	**I^2**	**Chi^2**	**P-value**	**OR**	**p-value**	**I^2**	**Chi^2**	**P-value**
CHAMPION PCI	0.90 (0.81-1.00)	0.04	65%	5.76	0.06	0.82 (0.72-0.93)	0.003	0%	0.50	0.48
CHAMPION PLATFORM	0.90 (0.81-1.00)	0.04	65%	5.76	0.06	0.91 (0.81-1.02)	0.12	82%	5.59	0.02
CHAMPION PHOENIX	0.90 (0.81-1.00)	0.04	65%	5.76	0.06	0.97 (0.85-1.11)	0.67	49%	1.97	0.16

Supplementary Table S9. Sensitivity analysis of the effect of $P2Y_{12}$ inhibitors on frequency of myocardial infarction in the comparison between cangrelor vs. clopidogrel

Study	Before excluding study					After excluding study				
	**OR**	**p-value**	**I^2**	**Chi^2**	**P-value**	**OR**	**p-value**	**I^2**	**Chi^2**	**P-value**
CHAMPION PCI	0.94 (0.77-1.14)	0.51	67%	6.15	0.05	0.85 (0.74-0.98)	0.02	0%	0.82	0.36
CHAMPION PLATFORM	0.94 (0.77-1.14)	0.51	67%	6.15	0.05	0.95 (0.69-1.29)	0.72	83%	6.04	0.01
CHAMPION PHOENIX	0.94 (0.77-1.14)	0.51	67%	6.15	0.05	1.02 (0.85-1.22)	0.85	45%	1.82	0.18

Supplementary Table S10. Node-splitting results of testing consistency for major adverse cardiovascular events (MACE) using random-effects model

Reference group	Comparison group	OR (95% CI) Combined direct and indirect evidence	OR (95% CI) Direct evidence	OR (95% CI) Indirect evidence	RoR Direct versus indirect	p value
Cangrelor	Clopidogrel	0.90 (0 .75-1.08)	0.90 (0 .76-1.06)	Not Applicable	Not Applicable	Not Applicable
Cangrelor	Prasugrel	1.12 (0.88-1.43)	Not Applicable	1.12 (0.88-1.43)	Not Applicable	Not Applicable
Cangrelor	Ticagrelor	1.01 (0.79-1.30)	Not Applicable	1.01 (0.79-1.30)	Not Applicable	Not Applicable
Clopidogrel	Prasugrel	1.25 (1.07-1.46)	1.21 (1.02-1.45)	1.39 (0.98-1.98)	0.87 (0.59-1.29)	0.487
Clopidogrel	Ticagrelor	1.13 (0.95-1.34)	1.17 (0.96-1.43)	1.02 (0.72-1.43)	1.15 (0.78-1.70)	0.487
Prasugrel	Ticagrelor	0.91 (0.74-1.10)	0.84 (0.63-1.12)	0.96 (0.74-1.26)	0.87 (0.59-1.29)	0.487

Supplementary Table S11. Node-splitting results of testing consistency for myocardial infarction using random-effects model

Reference group	Comparison group	OR (95% CI) Combined direct and indirect evidence	OR (95% CI) Direct evidence	OR (95% CI) Indirect evidence	RoR Direct versus indirect	p value
Cangrelor	Clopidogrel	0.94 (0.77-1.14)	0.94 (0.77-1.14)	Not Applicable	Not Applicable	Not Applicable
Cangrelor	Prasugrel	1.25 (0.96-1.62)	Not Applicable	1.25 (0.96-1.62)	Not Applicable	Not Applicable
Cangrelor	Ticagrelor	1.06 (0.80-1.41)	Not Applicable	1.06 (0.80-1.41)	Not Applicable	Not Applicable
Clopidogrel	Prasugrel	1.33 (1.12-1.59)	1.28 (1.05-1.55)	1.66 (1.07-2.59)	0.77 (0.47-1.24)	0.283
Clopidogrel	Ticagrelor	1.13 (0.92-1.40)	1.21 (0.95-1.54)	0.93 (0.61-1.41)	1.30 (0.80-2.12)	0.283
Prasugrel	Ticagrelor	0.85 (0.67-1.08)	0.73 (0.50-1.06)	0.95 (0.70-1.29)	0.77 (0.47-1.24)	0.283

Supplementary Table S12. Node-splitting results of testing consistency for stroke using random-effects model

Reference group	Comparison group	OR (95% CI) Combined direct and indirect evidence	OR (95% CI) Direct evidence	OR (95% CI) Indirect evidence	RoR Direct versus indirect	p value
Cangrelor	Clopidogrel	0.93 (0.43-1.99)	0.93 (0.43-1.99)	Not Applicable	Not Applicable	Not Applicable
Cangrelor	Prasugrel	0.97 (0.44-2.17)	Not Applicable	0.97 (0.44-2.17)	Not Applicable	Not Applicable
Cangrelor	Ticagrelor	0.85 (0.38-1.87)	Not Applicable	0.85 (0.38-1.87)	Not Applicable	Not Applicable
Clopidogrel	Prasugrel	1.05 (0.81-1.35)	1.08 (0.82-1.43)	0.91 (0.50-1.65)	1.19 (0.61-2.30)	0.606
Clopidogrel	Ticagrelor	0.91 (0.74-1.13)	0.89 (0.71-1.13)	1.06 (0.57-1.97)	0.84 (0.43-1.63)	0.606
Prasugrel	Ticagrelor	0.87 (0.64-1.18)	0.98 (0.57-1.71)	0.83 (0.58-1.19)	1.19 (0.61-2.30)	0.606

Supplementary Table S13. Node-splitting results of testing consistency for cardiovascular mortality using random-effects model

Reference group	Comparison group	OR (95% CI) Combined direct and indirect evidence	OR (95% CI) Direct evidence	OR (95% CI) Indirect evidence	RoR Direct versus indirect	p value
Cangrelor	Clopidogrel	1.00 (0.52-1.92)	1.00 (0.52-1.92)	Not Applicable	Not Applicable	Not Applicable
Cangrelor	Prasugrel	1.17 (0.60-2.29)	Not Applicable	1.17 (0.60-2.29)	Not Applicable	Not Applicable
Cangrelor	Ticagrelor	1.21 (0.62-2.36)	Not Applicable	1.21 (0.62-2.36)	Not Applicable	Not Applicable
Clopidogrel	Prasugrel	1.17 (1.03-1.34)	1.13 (0.98-1.31)	1.34 (1.01-1.79)	0.84 (0.61-1.16)	0.295
Clopidogrel	Ticagrelor	1.21 (1.08-1.37)	1.25 (1.10-1.42)	1.05 (0.79-1.41)	1.19 (0.86-1.63)	0.295
Prasugrel	Ticagrelor	1.03 (0.89-1.21)	0.93 (0.72-1.20)	1.10 (0.91-1.34)	0.84 (0.61-1.16)	0.295

Supplementary Table S14. Node-splitting results of testing consistency for definite or probable stent thrombosis using random-effects model

Reference group	Comparison group	OR (95% CI) Combined direct and indirect evidence	OR (95% CI) Direct evidence	OR (95% CI) Indirect evidence	RoR Direct versus indirect	p value
Cangrelor	Clopidogrel	0.80 (0.59-1.09)	0.80 (0.59-1.09)	Not Applicable	Not Applicable	Not Applicable
Cangrelor	Prasugrel	1.63 (1.09-2.43)	Not Applicable	1.63 (1.09-2.43)	Not Applicable	Not Applicable
Cangrelor	Ticagrelor	1.12 (0.76-1.63)	Not Applicable	1.12 (0.76-1.63)	Not Applicable	Not Applicable
Clopidogrel	Prasugrel	2.03 (1.58-2.62)	2.10 (1.58-2.79)	1.79 (1.03-3.14)	1.17 (0.62-2.19)	0.626
Clopidogrel	Ticagrelor	1.39 (1.12-1.74)	1.36 (1.07-1.73)	1.59 (0.89-2.84)	0.86 (0.46-1.60)	0.626
Prasugrel	Ticagrelor	0.69 (0.51-0.93)	0.76 (0.46-1.26)	0.65 (0.45-0.94)	1.17 (0.62-2.19)	0.626

Supplementary Table S15. Node-splitting results of testing consistency for Thrombolysis in Myocardial Infarction (TIMI) major bleeding using random-effects model

Reference group	Comparison group	OR (95% CI) Combined direct and indirect evidence	OR (95% CI) Direct evidence	OR (95% CI) Indirect evidence	RoR Direct versus indirect	p value
Cangrelor	Clopidogrel	1.01 (0.59-1.74)	1.01 (0.59-1.74)	Not Applicable	Not Applicable	Not Applicable
Cangrelor	Prasugrel	0.80 (0.45-1.43)	Not Applicable	0.80 (0.45-1.43)	Not Applicable	Not Applicable
Cangrelor	Ticagrelor	0.99 (0.57-1.72)	Not Applicable	0.99 (0.57-1.72)	Not Applicable	Not Applicable
Clopidogrel	Prasugrel	0.79 (0.64-0.98)	0.80 (0.64-0.98)	0.69 (0.19-2.47)	1.15 (0.32-4.19)	0.831
Clopidogrel	Ticagrelor	0.98 (0.88-1.09)	0.98 (0.88-1.09)	1.13 (0.31-4.08)	0.87 (0.24-3.16)	0.831
Prasugrel	Ticagrelor	1.23 (0.98-1.56)	1.41 (0.40-5.04)	1.23 (0.97-1.56)	1.15 (0.32-4.19)	0.831

Supplementary Table S16. Node-splitting results of testing consistency for Thrombolysis in Myocardial Infarction (TIMI) minor bleeding using random-effects model

Reference group	Comparison group	OR (95% CI) Combined direct and indirect evidence	OR (95% CI) Direct evidence	OR (95% CI) Indirect evidence	RoR Direct versus indirect	p value
Cangrelor	Clopidogrel	1.47 (1.01-2.16)	1.47 (1.01-2.16)	Not Applicable	Not Applicable	Not Applicable
Cangrelor	Prasugrel	1.03 (0.66-1.59)	Not Applicable	1.03 (0.66-1.59)	Not Applicable	Not Applicable
Cangrelor	Ticagrelor	1.35 (0.89-2.04)	Not Applicable	1.35 (0.89-2.04)	Not Applicable	Not Applicable
Clopidogrel	Prasugrel	0.70 (0.56-0.86)	0.70 (0.56-0.86)	Not Applicable	Not Applicable	Not Applicable
Clopidogrel	Ticagrelor	0.92 (0.78-1.08)	0.92 (0.78-1.08)	Not Applicable	Not Applicable	Not Applicable
Prasugrel	Ticagrelor	1.32 (1.01-1.72)	Not Applicable	1.32 (1.01-1.72)	Not Applicable	Not Applicable

Supplementary Table S17. Node-splitting results of testing consistency for all-cause mortality using random-effects model

Reference group	Comparison group	OR (95% CI) Combined direct and indirect evidence	OR (95% CI) Direct evidence	OR (95% CI) Indirect evidence	RoR Direct versus indirect	p value
Cangrelor	Clopidogrel	0.74 (0.47-1.16)	0.74 (0.47-1.16)	Not Applicable	Not Applicable	Not Applicable
Cangrelor	Prasugrel	0.81 (0.50-1.32)	Not Applicable	0.81 (0.50-1.32)	Not Applicable	Not Applicable
Cangrelor	Ticagrelor	0.85 (0.53-1.38)	Not Applicable	0.85 (0.53-1.38)	Not Applicable	Not Applicable
Clopidogrel	Prasugrel	1.10 (0.94-1.29)	1.04 (0.87-1.25)	1.38 (0.97-1.96)	0.75 (0.51-1.12)	0.162
Clopidogrel	Ticagrelor	1.16 (0.98-1.36)	1.22 (1.02-1.47)	0.92 (0.65-1.31)	1.32 (0.89-1.96)	0.162
Prasugrel	Ticagrelor	1.05 (0.86-1.27)	0.89 (0.66-1.20)	1.18 (0.91-1.52)	0.75 (0.51-1.12)	0.162

Supplementary Table S18. Effect of different P2Y12 inhibitors on frequencies of all outcomes in ACS patients established by network meta-analysis using fixed-effects models

	Clopidogrel	Prasugrel	Ticagrelor	Cangrelor
**MACE**				
Clopidogrel	1.00	**0.81 (0.75-0.88)**	**0.87 (0.80-0.94)**	**0.90 (0.81-1.00)**
Prasugrel	**1.24 (1.14-1.34)**	1.00	1.07 (0.96-1.19)	1.11 (0.98-1.27)
Ticagrelor	**1.15 (1.06-1.25)**	0.93 (0.84-1.04)	1.00	1.04 (0.91-1.19)
Cangrelor	**1.11 (1.00-1.23)**	0.90 (0.79-1.02)	0.96 (0.84-1.10)	1.00
**Myocardial infarction**				
Clopidogrel	1.00	**0.76 (0.69-0.83)**	**0.87 (0.78-0.97)**	0.95 (0.85-1.05)
Prasugrel	**1.32 (1.20-1.45)**	1.00	1.14 (1.00-1.30)	**1.24 (1.08-1.44)**
Ticagrelor	**1.16 (1.04-1.29)**	0.88 (0.77-1.00)	1.00	1.09 (0.94-1.27)
Cangrelor	1.06 (0.95-1.18)	**0.80 (0.70-0.93)**	0.92 (0.79-1.07)	1.00
**Stroke**				
Clopidogrel	1.00	0.96 (0.74-1.23)	1.10 (0.88-1.36)	1.08 (0.49-2.37)
Prasugrel	1.05 (0.81-1.35)	1.00	1.15 (0.85-1.55)	1.13 (0.49-2.59)
Ticagrelor	0.91 (0.74-1.13)	0.87 (0.64-1.18)	1.00	0.98 (0.43-2.23)
Cangrelor	0.93 (0.42-2.05)	0.89 (0.39-2.04)	1.02 (0.45-2.31)	1.00
**Cardiovascular mortality**				
Clopidogrel	1.00	**0.85 (0.75-0.97)**	**0.82 (0.73-0.93)**	1.00 (0.52-1.92)
Prasugrel	**1.17 (1.03-1.34)**	1.00	0.97 (0.83-1.13)	1.17 (0.60-2.29)
Ticagrelor	**1.21 (1.08-1.37)**	1.03 (0.89-1.21)	1.00	1.21 (0.62-2.36)
Cangrelor	1.00 (0.52-1.92)	0.85 (0.44-1.66)	0.82 (0.42-1.60)	1.00
**Definite or probable stent thrombosis**				
Clopidogrel	1.00	**0.49 (0.38-0.63)**	**0.72 (0.57-0.90)**	**0.59 (0.43-0.81)**
Prasugrel	**2.03 (1.58-2.62)**	1.00	**1.46 (1.08-1.97)**	1.20 (0.80-1.81)

Ticagrelor	**1.39 (1.12-1.74)**	**0.69 (0.51-0.93)**	1.00	0.83 (0.56-1.22)
Cangrelor	**1.69 (1.23-2.32)**	0.83 (0.55-1.25)	1.21 (0.82-1.78)	1.00
**All-cause mortality**				
Clopidogrel	1.00	0.91 (0.80-1.03)	**0.83 (0.74-0.93)**	0.76 (0.48-1.20)
Prasugrel	1.10 (0.97-1.25)	1.00	0.92 (0.79-1.07)	0.84 (0.52-1.35)
Ticagrelor	**1.20 (1.07-1.35)**	**1.09 (0.93-1.27)**	1.00	0.91 (0.56-1.46)
Cangrelor	1.32 (0.83-2.10)	1.20 (0.74-1.93)	1.10 (0.68-1.77)	1.00
**All major bleeding**				
Clopidogrel	1.00	**1.24 (1.05-1.48)**	1.07 (0.97-1.19)	1.01 (0.59-1.74)
Prasugrel	**0.80 (0.68-0.95)**	1.00	0.86 (0.72-1.03)	0.81 (0.46-1.43)
Ticagrelor	0.93 (0.84-1.03)	1.16 (0.97-1.39)	1.00	0.94 (0.55-1.63)
Cangrelor	0.99 (0.58-1.69)	1.23 (0.70-2.16)	1.06 (0.61-1.83)	1.00
**TIMI major bleeding**				
Clopidogrel	1.00	**1.36 (1.11-1.66)**	1.02 (0.92-1.14)	1.01 (0.59-1.74)
Prasugrel	**0.73 (0.60-0.90)**	1.00	**0.75 (0.60-0.95)**	0.75 (0.42-1.32)
Ticagrelor	0.98 (0.88-1.09)	**1.33 (1.06-1.67)**	1.00	0.99 (0.57-1.71)
Cangrelor	0.99 (0.58-1.69)	1.34 (0.76-2.38)	1.01 (0.58-1.75)	1.00
**Non-CABG-related TIMI major bleeding**				
Clopidogrel	1.00	**1.26 (1.02-1.55)**	**1.25 (1.03-1.52)**	1.00 (0.29-3.45)
Prasugrel	**0.80 (0.64-0.98)**	1.00	0.99 (0.75-1.33)	0.80 (0.23-2.80)
Ticagrelor	**0.80 (0.66-0.97)**	1.01 (0.76-1.34)	1.00	0.80 (0.23-2.81)
Cangrelor	1.00 (0.29-3.46)	1.26 (0.36-4.42)	1.25 (0.36-4.38)	1.00
**CABG-related TIMI major bleeding***				
Clopidogrel	1.00	**1.32 (1.03-1.69)**	0.93 (0.81-1.06)	NA
Prasugrel	**0.76 (0.59-0.97)**	1.00	**0.70 (0.53-0.93)**	NA
Ticagrelor	1.08 (0.95-1.23)	**1.42 (1.07-1.89)**	1.00	NA
Cangrelor	NA	NA	NA	1.00

**TIMI minor bleeding**				
Clopidogrel	1.00	**1.44 (1.16-1.77)**	1.09 (0.93-1.28)	**1.47 (1.01-2.16)**
Prasugrel	**0.70 (0.56-0.86)**	1.00	**0.76 (0.58-0.99)**	1.03 (0.66-1.59)
Ticagrelor	0.92 (0.78-1.08)	**1.32 (1.01-1.72)**	1.00	1.35 (0.89-2.04)
Cangrelor	**0.68 (0.46-0.99)**	0.97 (0.63-1.50)	0.74 (0.49-1.12)	1.00

Abbreviations used in Supplementary Table S18: ACS = acute coronary syndrome; CABG = coronary artery bypass grafting; MACE = major cardiovascular events; NA = not applicable; TIMI = Thrombolysis in Myocardial Infarction.

Results are the Odds Ratios (95% Confidence Interval) in the column-defining therapy compared with the Odds Ratios in the row-defining therapy. For efficacy and safety, Odds Ratio <1 favours the column-defining therapy. Significant results are shown in bold.

* CABG-related TIMI major bleeding was not reported in any comparison involving cangrelor.

Supplementary Table S19. Estimates of risk of outcomes between different P2Y12 inhibitors using a Bayesian framework with non-informative priors

	Clopidogrel	Prasugrel	Ticagrelor	Cangrelor
**MACE**				
Clopidogrel	1.00	**0.80 (0.68-0.95)**	0.88 (0.73-1.00)	0.90 (0.74-1.10)
Prasugrel	**1.20 (1.10-1.50)**	1.00	1.10 (0.88-1.30)	1.10 (0.87-1.50)
Ticagrelor	1.10 (0.96-1.40)	0.92 (0.74-1.10)	1.00	1.00 (0.80-1.40)
Cangrelor	1.10 (0.90-1.30)	0.89 (0.68-1.10)	0.97 (0.74-1.20)	1.00
**Myocardial infarction**				
Clopidogrel	1.00	**0.75 (0.61-0.91)**	0.87 (0.68-1.10)	0.94 (0.75-1.20)
Prasugrel	**1.30 (1.10-1.60)**	1.00	1.20 (0.89-1.50)	1.30 (0.95-1.70)
Ticagrelor	1.20 (0.93-1.50)	0.87 (0.68-1.10)	1.00	1.10 (0.80-1.50)
Cangrelor	1.10 (0.86-1.30)	0.80 (0.60-1.10)	0.92 (0.66-1.30)	1.00
**Stroke**				
Clopidogrel	1.00	0.94 (0.68-1.30)	1.10 (0.76-1.40)	1.10 (0.49-2.60)
Prasugrel	1.10 (0.77-1.50)	1.00	1.10 (0.76-1.60)	1.20 (0.49-2.80)
Ticagrelor	0.94 (0.72-1.30)	0.90 (0.62-1.30)	1.00	1.00 (0.45-2.60)
Cangrelor	0.91 (0.39-2.00)	0.85 (0.35-2.10)	0.96 (0.39-2.20)	1.00
**Cardiovascular mortality**				
Clopidogrel	1.00	0.87 (0.73-1.00)	**0.82 (0.69-0.99)**	1.00 (0.51-2.00)
Prasugrel	1.20 (0.98-1.40)	1.00	0.95 (0.77-1.20)	1.20 (0.57-2.30)
Ticagrelor	**1.20 (1.00-1.40)**	1.10 (0.84-1.30)	1.00	1.20 (0.61-2.40)
Cangrelor	0.98 (0.51-2.00)	0.85 (0.43-1.80)	0.80 (0.41-1.70)	1.00
**Definite or probable stent thrombosis**				
Clopidogrel	1.00	**0.49 (0.34-0.70)**	**0.70 (0.47-0.96)**	**0.58 (0.38-0.85)**
Prasugrel	**2.00 (1.40-2.90)**	1.00	1.40 (0.93-2.10)	1.20 (0.66-2.00)
Ticagrelor	**1.40 (1.00-2.10)**	0.71 (0.48-1.10)	1.00	0.83 (0.49-1.40)
Cangrelor	**1.70 (1.20-2.60)**	0.85 (0.49-1.50)	1.20 (0.70-2.00)	1.00

**All-cause mortality**				
Clopidogrel	1.00	0.92 (0.74-1.20)	0.88 (0.73-1.10)	0.72 (0.42-1.20)
Prasugrel	1.10 (0.85-1.30)	1.00	0.96 (0.75-1.20)	0.78 (0.44-1.30)
Ticagrelor	1.10 (0.90-1.40)	1.00 (0.81-1.30)	1.00	0.81 (0.46-1.40)
Cangrelor	1.40 (0.86-2.40)	1.30 (0.76-2.30)	1.20 (0.74-2.20)	1.00
**All major bleeding**				
Clopidogrel	1.00	1.20 (0.96-1.60)	1.10 (0.92-1.50)	0.98 (0.54-1.80)
Prasugrel	0.81 (0.63-1.00)	1.00	0.92 (0.70-1.30)	0.79 (0.42-1.50)
Ticagrelor	0.88 (0.69-1.10)	1.10 (0.78-1.40)	1.00	0.86 (0.45-1.70)
Cangrelor	1.00 (0.55-1.90)	1.30 (0.65-2.40)	1.20 (0.60-2.20)	1.00
**TIMI major bleeding**				
Clopidogrel	1.00	1.30 (0.99-1.70)	1.00 (0.75-1.30)	1.00 (0.57-1.80)
Prasugrel	0.76 (0.58-1.00)	1.00	0.77 (0.53-1.10)	0.76 (0.40-1.40)
Ticagrelor	0.98 (0.78-1.30)	1.30 (0.89-1.90)	1.00	0.99 (0.53-1.80)
Cangrelor	0.99 (0.56-1.70)	1.30 (0.71-2.50)	1.00 (0.54-1.90)	1.00
**TIMI minor bleeding**				
Clopidogrel	1.00	**1.50 (1.10-2.20)**	1.10 (0.85-1.60)	1.60 (1.00-2.40)
Prasugrel	**0.67 (0.46-0.87)**	1.00	0.75 (0.47-1.20)	1.00 (0.59-1.70)
Ticagrelor	0.88 (0.62-1.20)	1.30 (0.84-2.20)	1.00	1.40 (0.78-2.40)
Cangrelor	0.64 (0.41-1.00)	0.99 (0.57-1.70)	0.74 (0.42-1.30)	1.00

Abbreviations used in Supplementary Table S19: ACS = acute coronary syndrome; CABG = coronary artery bypass grafting; MACE = major cardiovascular events; TIMI = Thrombolysis in Myocardial Infarction.

Results are the Odds Ratios (95% Confidence Interval) in the column-defining therapy compared with the Odds Ratios in the row-defining therapy. For efficacy and safety, Odds Ratio <1 favours the column-defining therapy. Significant results are shown in bold.

Supplementary Table S20. Analysis of funnel plots in Thrombolysis in Supplementary Figs. S2-S9 to assess publication bias

Suppl. Fig	Trim and fill: Before trimming, Observed Point estimate (LL-UL)	After trimming: Trimming direction	After trimming: Adjusted Point estimate (LL-UL)
S2A	0.830 (0.763-0.904)	Right	0.839 (0.773-0.910)
S2B	0.840 (0.637-1.107)	Right	0.901 (0.672-1.208)
S2C	0.898 (0.749-1.077)	Not Applicable	0.898 (0.749-1.077)
S2D*	Not Applicable	Not Applicable	Not Applicable
S3A	0.882 (0.764-1.020)	Right	0.930 (0.828-1.045)
S3B	0.819 (0.635-1.056)	Not Applicable	0.819 (0.635-1.056)
S3C	Not Applicable	Not Applicable	Not Applicable
S3D*	Not Applicable	Not Applicable	Not Applicable
S4A	0.799 (0.706-0.860)	Right	0.782 (0.710-0.862)
S4B	0.808 (0.586-1.111)	Right	0.905 (0.638-1.283)
S4C	0.936 (0.762-1.151)	Not Applicable	0.936 (0.762-1.151)
S4D*	Not Applicable	Not Applicable	Not Applicable
S5A*	Not Applicable	Not Applicable	Not Applicable
S5B*	Not Applicable	Not Applicable	Not Applicable
S5C	0.689 (0.394-1.202)	Not Applicable	0.689 (0.394-1.202)
S5D*	Not Applicable	Not Applicable	Not Applicable
S6A	0.957 (0.832-1.110)	Left	0.954 (0.831-1.097)
S6B	0.830 (0.668-1.030)	Not Applicable	0.830 (0.668-1.030)
S6C	0.727 (0.431-1.225)	Not Applicable	0.727 (0.431-1.225)
S6D*	Not Applicable	Not Applicable	Not Applicable
S7A	0.926 (0.700-1.225)	Left	0.917 (0.694-1.211)
S7B	1.119 (0.889-1.409)	Right	1.125 (0.825-1.536)

S7C*	Not Applicable	Not Applicable	Not Applicable
S7D*	Not Applicable	Not Applicable	Not Applicable
S8A	1.358 (1.108-1.665)	Right	1.417 (1.112-1.807)
S8B	1.025 (0.918-1.143)	Not Applicable	1.025 (0.918-1.143)
S8C	0.962 (0.505-1.832)	Not Applicable	0.962 (0.505-1.832)
S8D*	Not Applicable	Not Applicable	Not Applicable
S9A	1.440 (1.168-1.776)	Left	1.369 (1.124-1.668)
S9B	1.135 (0.830-1.554)	Left	1.085 (0.770-1.528)
S9C	1.474 (1.007-2.158)	Not Applicable	1.474 (1.007-2.158)
S9D*	Not Applicable	Not Applicable	Not Applicable
S10A	1.210 (0.982-1.490)	Right	1.222 (0.994-1.503)
S10B	1.048 (0.942-1.167)	Left	1.047 (0.941-1.165)
S10C	0.962 (0.505-1.832)	Not Applicable	0.962 (0.505-1.832)
S10D*	Not Applicable	Not Applicable	Not Applicable

Abbreviations used in Supplementary Table S20: LL = Lower limit; Suppl. Fig = Supplementary Figure; UL = Upper limit.

* Less than 3 studies in each comparison assessed.

Supplementary Table S21. Effect of oral P2Y12 inhibitors on frequencies of clinical outcomes in ACS patients established by network meta-analysis

	Clopidogrel	Prasugrel	Ticagrelor
**MACE**			
Clopidogrel	1.00	**0.80 (0.68-0.94)**	0.89 (0.75-1.05)
Prasugrel	**1.25 (1.06-1.46)**	1.00	1.10 (0.91-1.34)
Ticagrelor	1.13 (0.95-1.34)	0.91 (0.74-1.10)	1.00
**Myocardial infarction**			
Clopidogrel	1.00	**0.75 (0.63-0.89)**	0.88 (0.72-1.08)
Prasugrel	**1.33 (1.12-1.58)**	1.00	1.17 (0.93-1.48)
Ticagrelor	1.13 (0.93-1.39)	0.85 (0.68-1.07)	1.00
**Stroke**			
Clopidogrel	1.00	0.96 (0.74-1.23)	1.10 (0.88-1.36)
Prasugrel	1.05 (0.81-1.35)	1.00	1.15 (0.85-1.55)
Ticagrelor	0.91(0.74-1.13)	0.87 (0.64-1.18)	1.00
**Cardiovascular mortality**			
Clopidogrel	1.00	**0.85 (0.75-0.97)**	**0.82 (0.73-0.93)**
Prasugrel	**1.17 (1.03-1.34)**	1.00	0.97 (0.83-1.13)
Ticagrelor	**1.21 (1.08-1.37)**	1.03 (0.89-1.21)	1.00
**Definite or probable stent thrombosis**			
Clopidogrel	1.00	**0.49 (0.38-0.63)**	**0.72 (0.57-0.90)**
Prasugrel	**2.03 (1.58-2.62)**	1.00	**1.46 (1.08-1.97)**
Ticagrelor	**1.39 (1.12-1.74)**	**0.69 (0.51-0.93)**	1.00
**All-cause mortality**			
Clopidogrel	1.00	0.91 (0.78-1.05)	**0.86 (0.74-0.99)**
Prasugrel	1.10 (0.95-1.28)	1.00	0.95 (0.79-1.14)
Ticagrelor	**1.17 (1.01-1.35)**	1.06 (0.88-1.27)	1.00
**All major bleeding**			
Clopidogrel	1.00	**1.24 (1.05-1.48)**	1.07 (0.97-1.19)
Prasugrel	**0.80 (0.68-0.95)**	1.00	0.86 (0.72-1.03)
Ticagrelor	0.93 (0.84-1.03)	1.16 (0.97-1.39)	1.00
**TIMI major bleeding**			
Clopidogrel	1.00	**1.36 (1.11-1.66)**	1.02 (0.92-1.14)
Prasugrel	**0.73 (0.60-0.90)**	1.00	**0.75 (0.60-0.95)**
Ticagrelor	0.98 (0.88-1.09)	**1.33 (1.06-1.67)**	1.00

Abbreviations used in Supplementary Table S21: ACS = acute coronary syndrome; MACE = major cardiovascular events; TIMI = Thrombolysis in Myocardial Infarction.

Results are the Odds Ratios (95% Confidence Interval) in the column-defining therapy compared with the Odds Ratios in the row-defining therapy. For efficacy and safety, Odds Ratio <1 favours the column-defining therapy. Significant results are shown in bold.

Supplementary Table S22. Effect of P2Y12 inhibitors on frequencies of clinical outcomes in ACS patients including the POPular AGE trial

	Clopidogrel	Prasugrel	Ticagrelor	Cangrelor
**MACE**				
Clopidogrel	1.00	**0.81 (0.69-0.94)**	0.91 (0.77-1.06)	0.90 (0.75-1.07)
Prasugrel	**1.24 (1.16-1.45)**	1.00	1.12 (0.93-1.36)	1.11 (0.88-1.41)
Ticagrelor	1.10 (0.94-1.29)	0.89 (0.74-1.07)	1.00	0.99 (0.78-1.26)
Cangrelor	1.12 (0.93-1.33)	0.90 (0.71-1.14)	1.01 (0.80-1.28)	1.00
**Myocardial infarction**				
Clopidogrel	1.00	**0.75 (0.64-0.89)**	0.90 (0.74-1.08)	0.94 (0.78-1.13)
Prasugrel	**1.33 (1.12-1.57)**	1.00	1.19 (0.95-1.48)	1.24 (0.97-1.59)
Ticagrelor	1.12 (0.93-1.34)	0.84 (0.68-1.05)	1.00	1.05 (0.81-1.36)
Cangrelor	1.07 (0.89-1.28)	0.80 (0.63-1.03)	0.96 (0.74-1.24)	1.00
**Stroke**				
Clopidogrel	1.00	0.96 (0.75-1.24)	1.12 (0.91-1.39)	1.08 (0.49-2.37)
Prasugrel	1.04 (0.81-1.34)	1.00	1.17 (0.86-1.58)	1.12 (0.49-2.57)
Ticagrelor	0.89 (0.72-1.10)	0.86 (0.63-1.16)	1.00	0.96 (0.42-2.18)
Cangrelor	0.93 (0.42-2.05)	0.89 (0.39-2.05)	1.04 (0.46-2.36)	1.00
**Cardiovascular mortality**				
Clopidogrel	1.00	**0.85 (0.75-0.97)**	**0.82 (0.73-0.93)**	1.00 (0.52-1.92)
Prasugrel	**1.17 (1.03-1.34)**	1.00	0.97 (0.83-1.13)	1.17 (0.60-2.29)
Ticagrelor	**1.21 (1.08-1.37)**	1.03 (0.89-1.21)	1.00	1.21 (0.62-2.36)
Cangrelor	1.00 (0.52-1.92)	0.85 (0.44-1.66)	0.82 (0.42-1.60)	1.00
**Definite or probable stent thrombosis**				
Clopidogrel	1.00	**0.49 (0.38-0.63)**	**0.71 (0.57-0.89)**	**0.59 (0.43-0.81)**
Prasugrel	**2.04 (1.58-2.63)**	1.00	**1.45 (1.07-1.95)**	1.21 (0.81-1.81)
Ticagrelor	**1.41 (1.13-1.76)**	**0.69 (0.51-0.93)**	1.00	0.84 (0.57-1.23)
Cangrelor	**1.69 (1.23-2.32)**	0.83 (0.55-1.24)	1.20 (0.81-1.76)	1.00
**All-cause mortality**				
Clopidogrel	1.00	0.91 (0.77-1.07)	0.87 (0.75-1.02)	0.75 (0.47-1.21)
Prasugrel	1.10 (0.94-1.30)	1.00	0.96 (0.79-1.17)	0.83 (0.50-1.37)
Ticagrelor	1.15 (0.98-1.34)	1.04 (0.85-1.26)	1.00	0.86 (0.52-1.42)
Cangrelor	1.33 (0.83-2.14)	1.20 (0.73-1.90)	1.16 (0.70-1.91)	1.00
**All major bleeding**				
Clopidogrel	1.00	**1.25 (1.01-1.55)**	1.17 (0.98-1.39)	1.00 (0.58-1.75)
Prasugrel	**0.80 (0.64-0.99)**	1.00	0.93 (0.74-1.18)	0.80 (0.44-1.45)
Ticagrelor	0.85 (0.72-1.02)	1.17 (0.85-1.36)	1.00	0.86 (0.48-1.53)
Cangrelor	1.00 (0.57-1.74)	1.25 (0.69-2.27)	1.17 (0.65-2.09)	1.00
**TIMI major bleeding**				
Clopidogrel	1.00	**1.34 (1.06-1.69)**	1.08 (0.89-1.27)	1.01 (0.58-1.74)
Prasugrel	**0.75 (0.59-0.94)**	1.00	0.80 (0.59-1.06)	0.75 (0.41-1.37)
Ticagrelor	0.94 (0.79-1.12)	1.26 (0.94-1.68)	1.00	0.95 (0.53-1.69)

Cangrelor	0.99 (0.57-1.72)	1.33 (0.73-2.41)	1.06 (0.59-1.88)	1.00
**TIMI minor bleeding**				
Clopidogrel	1.00	**1.44 (1.16-1.77)**	1.10 (0.94-1.29)	**1.47 (1.01-2.16)**
Prasugrel	**0.70 (0.56-0.86)**	1.00	**0.77 (0.58-1.00) (p = 0.048)**	1.03 (0.66-1.59)
Ticagrelor	0.91 (0.78-1.06)	**1.30 (1.00-1.69) (p = 0.048)**	1.00	1.34 (0.89-2.02)
Cangrelor	**0.68 (0.46-0.99)**	0.97 (0.63-1.50)	0.74 (0.49-1.13)	1.00

Abbreviations used in Supplementary Table S22: ACS = acute coronary syndrome; CABG = coronary artery bypass grafting; MACE = major cardiovascular events; NA = not applicable; TIMI = Thrombolysis in Myocardial Infarction.

Results are the Odds Ratios (95% Confidence Interval) in the column-defining therapy compared with the Odds Ratios in the row-defining therapy. For efficacy and safety, Odds Ratio <1 favours the column-defining therapy. Significant results are shown in bold.

[*] CABG-related TIMI major bleeding was not reported in any comparison involving cangrelor.

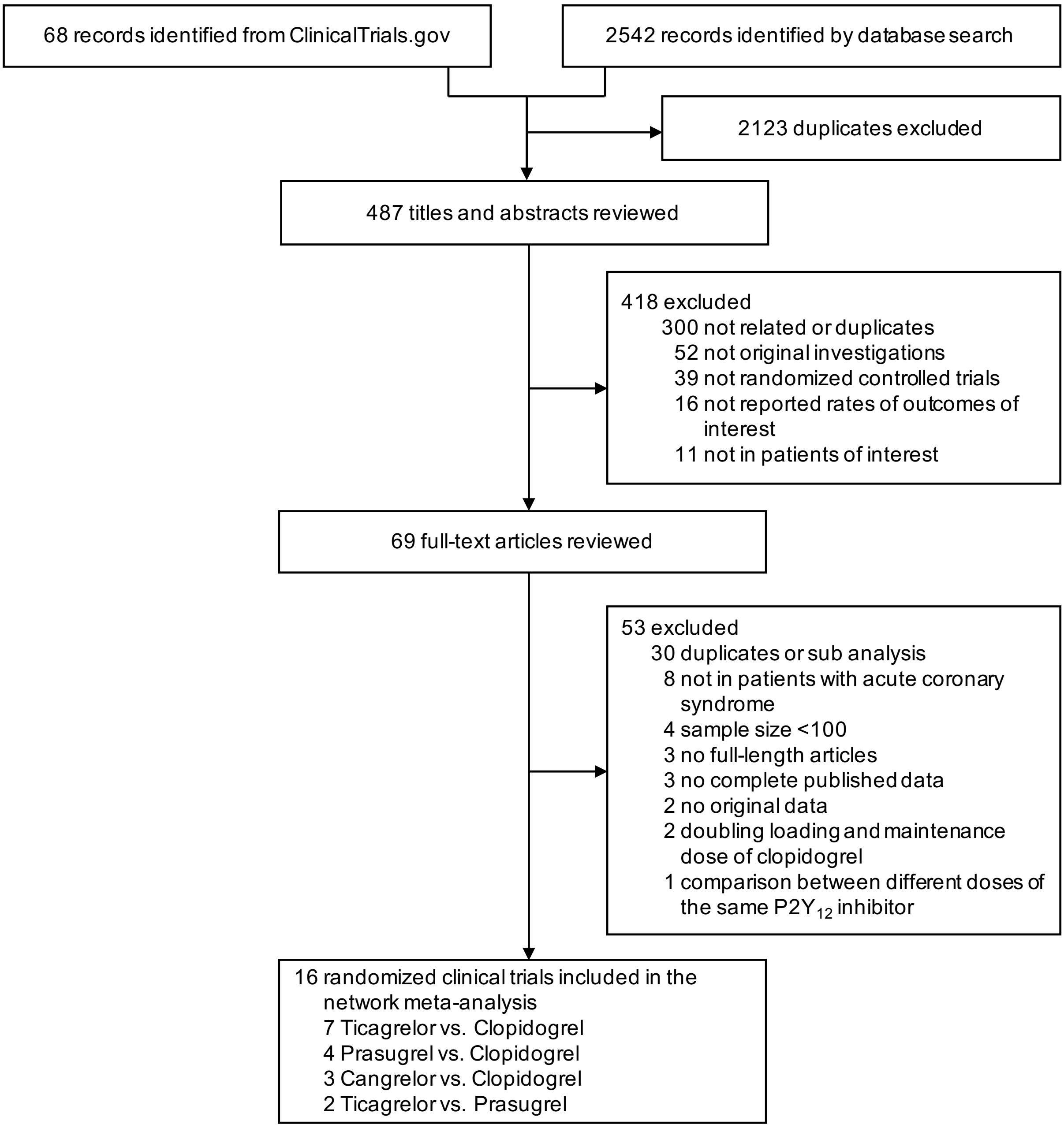

Supplementary Fig. S1. Flow diagram of scientific literature search and study selection.

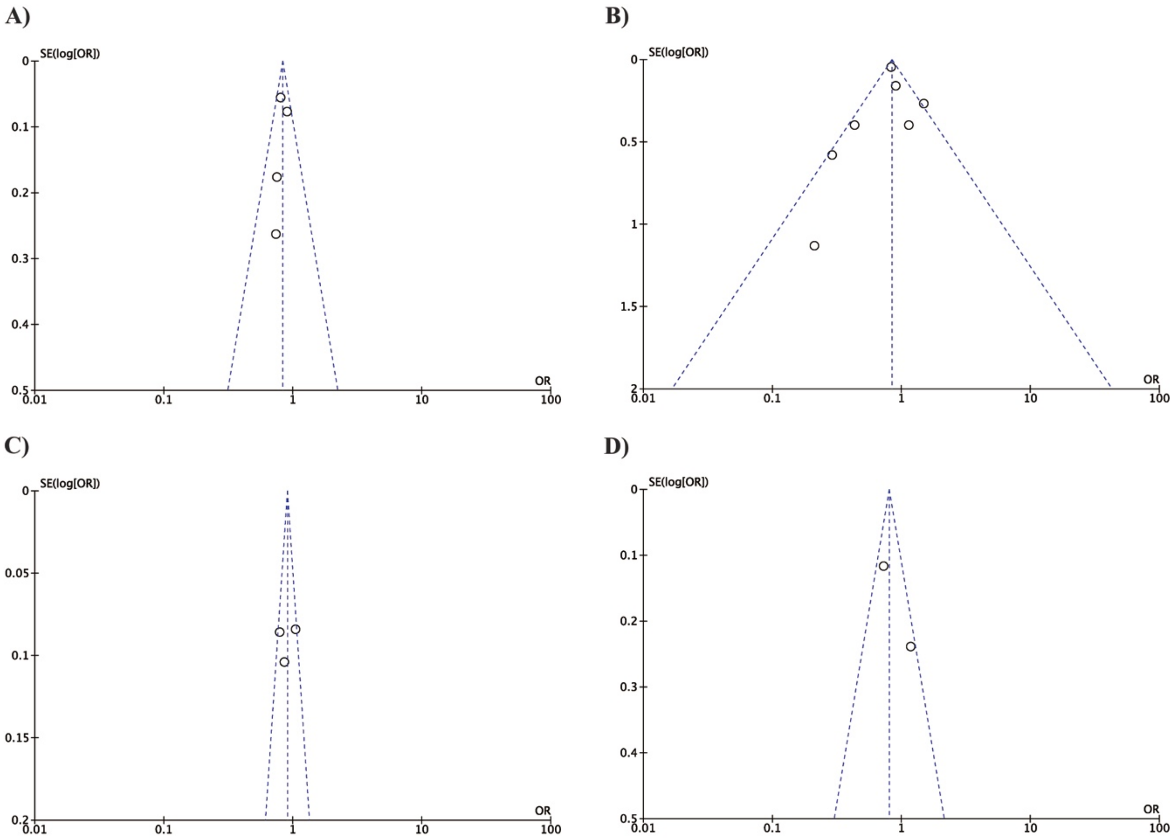

Supplementary Fig. S2. Funnel plot showing publication bias for the effect of different P2Y12 inhibitors on frequencies of major adverse cardiovascular events (MACE) in patients with acute coronary syndrome

(A) prasugrel versus clopidogrel. (B) ticagrelor versus clopidogrel. C) cangrelor versus clopidogrel. D) prasugrel versus ticagrelor.

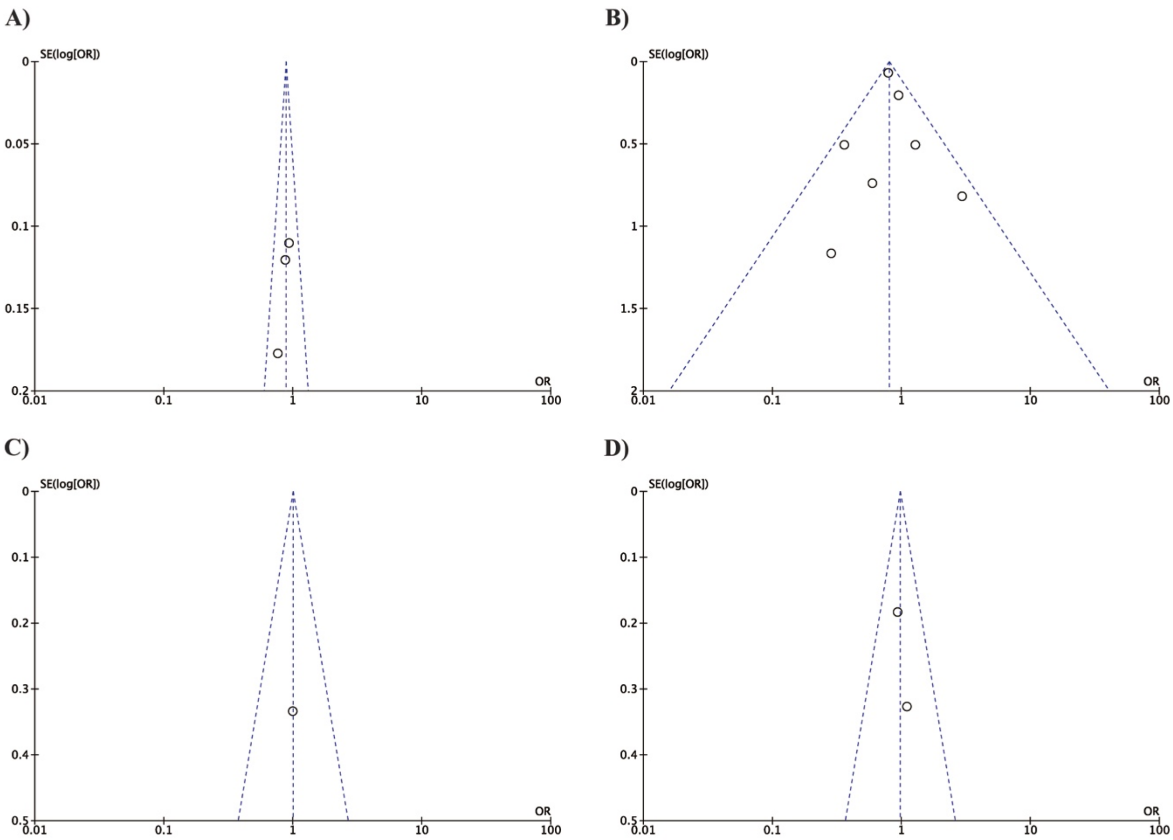

Supplementary Fig. S3. Funnel plot showing publication bias for the effect of different $P2Y_{12}$ inhibitors on frequencies of cardiovascular mortality in patients with acute coronary syndrome

(A) prasugrel versus clopidogrel. (B) ticagrelor versus clopidogrel. C) cangrelor versus clopidogrel. D) prasugrel versus ticagrelor.

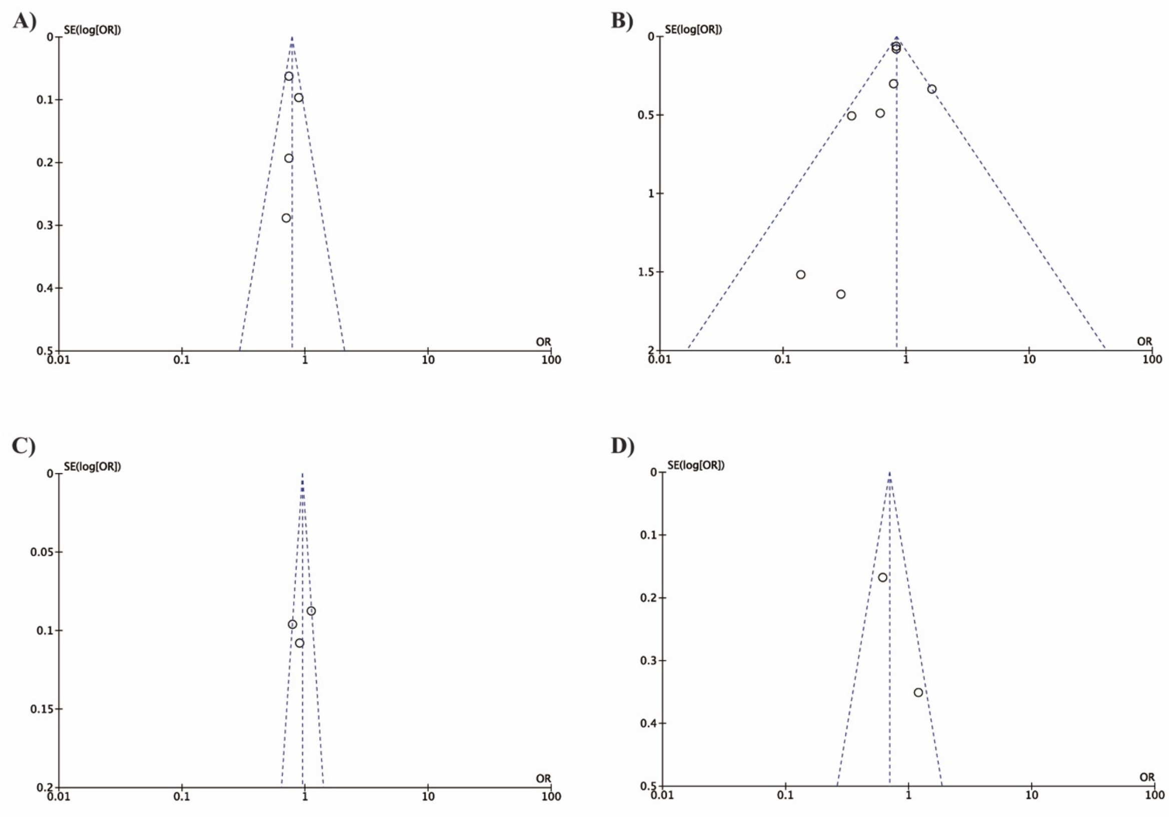

Supplementary Fig. S4. Funnel plot showing publication bias for the effect of different P2Y12 inhibitors on frequencies of myocardial infarction in patients with acute coronary syndrome

(A) prasugrel versus clopidogrel. (B) ticagrelor versus clopidogrel. C) cangrelor versus clopidogrel. D) prasugrel versus ticagrelor.

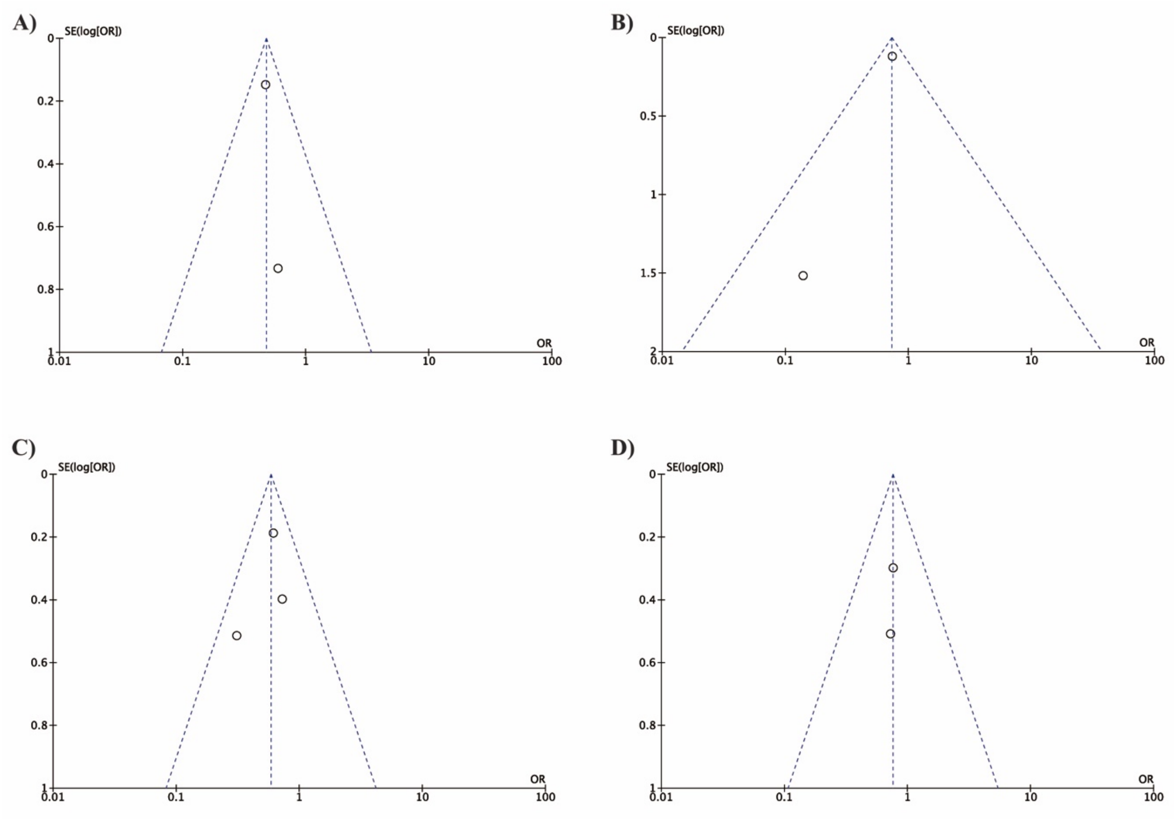

Supplementary Fig S5. Funnel plot showing publication bias for the effect of different $P2Y_{12}$ inhibitors on frequencies of definite and probable stent thrombosis in patients with acute coronary syndrome

(A) prasugrel versus clopidogrel. (B) ticagrelor versus clopidogrel. C) cangrelor versus clopidogrel. D) prasugrel versus ticagrelor.

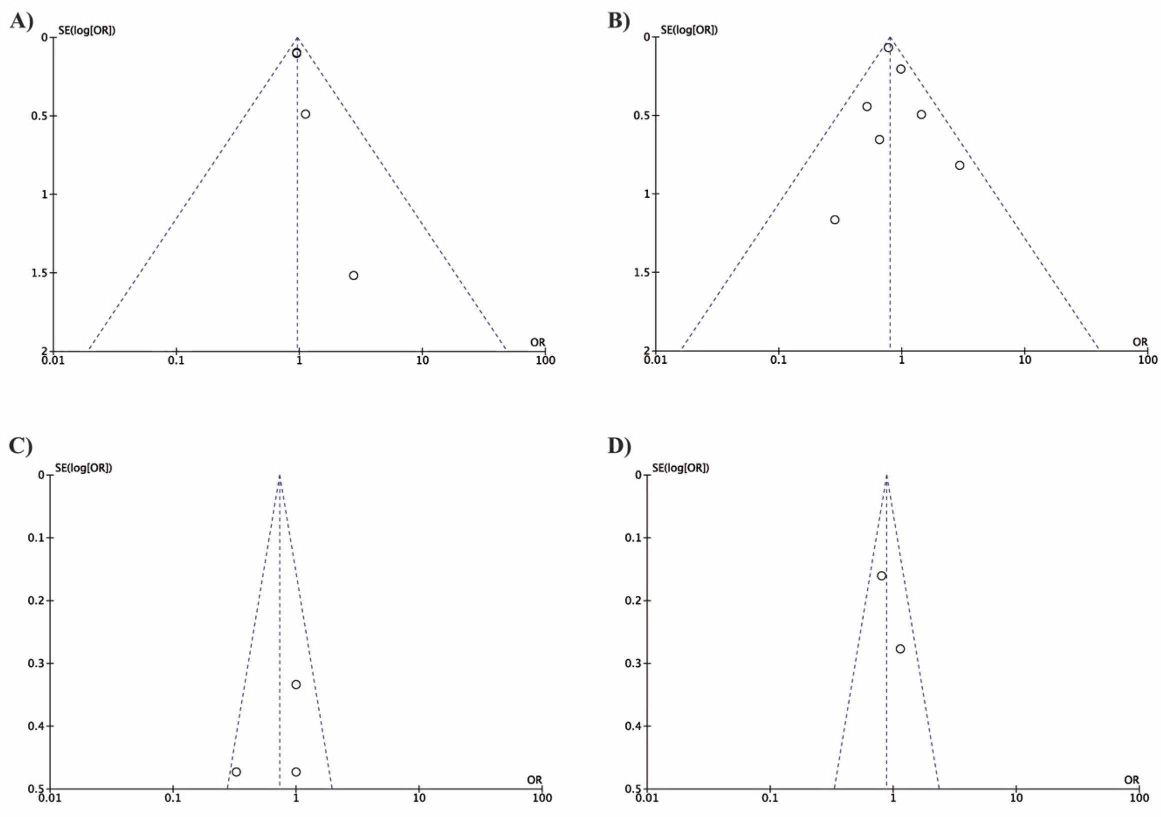

Supplementary Fig. S6. Funnel plot showing publication bias for the effect of different P2Y$_{12}$ inhibitors on frequencies of all-cause mortality in patients with acute coronary syndrome

(A) prasugrel versus clopidogrel. (B) ticagrelor versus clopidogrel. C) cangrelor versus clopidogrel. D) prasugrel versus ticagrelor.

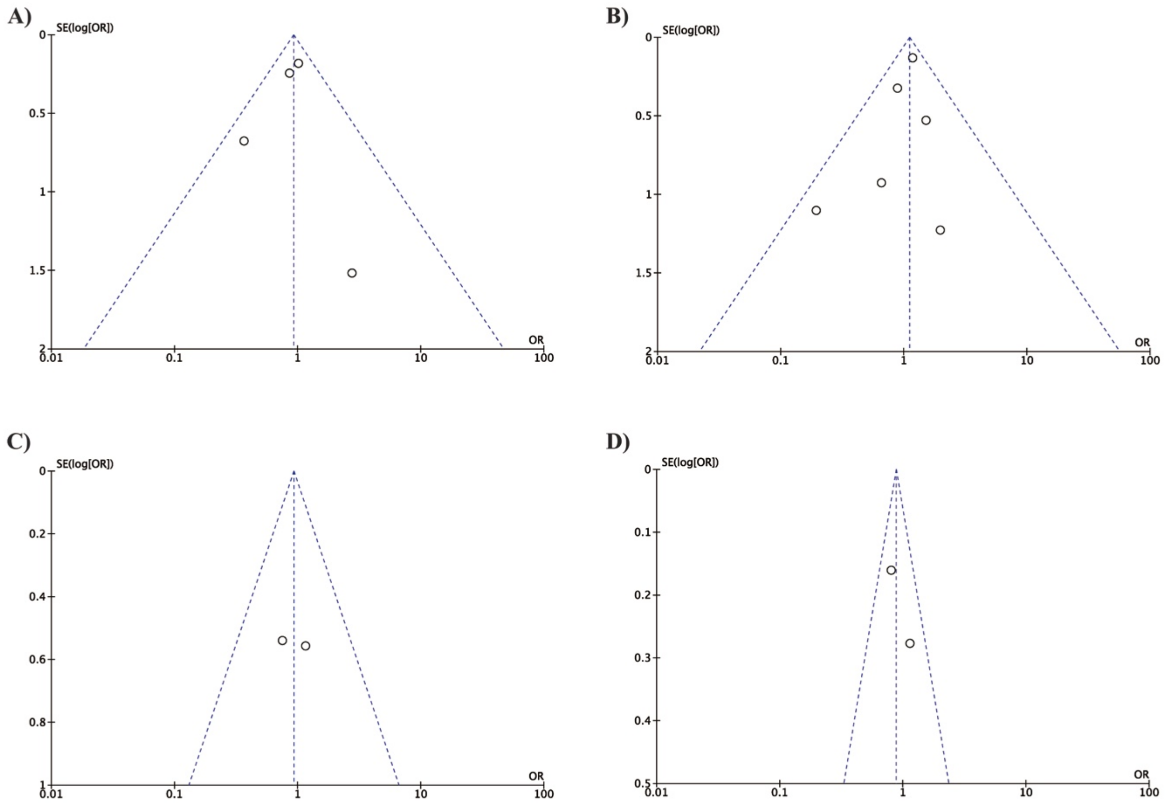

Supplementary Fig. S7. Funnel plot showing publication bias for the effect of different P2Y$_{12}$ inhibitors on frequencies of stroke in patients with acute coronary syndrome

(A) prasugrel versus clopidogrel. (B) ticagrelor versus clopidogrel. C) cangrelor versus clopidogrel. D) prasugrel versus ticagrelor.

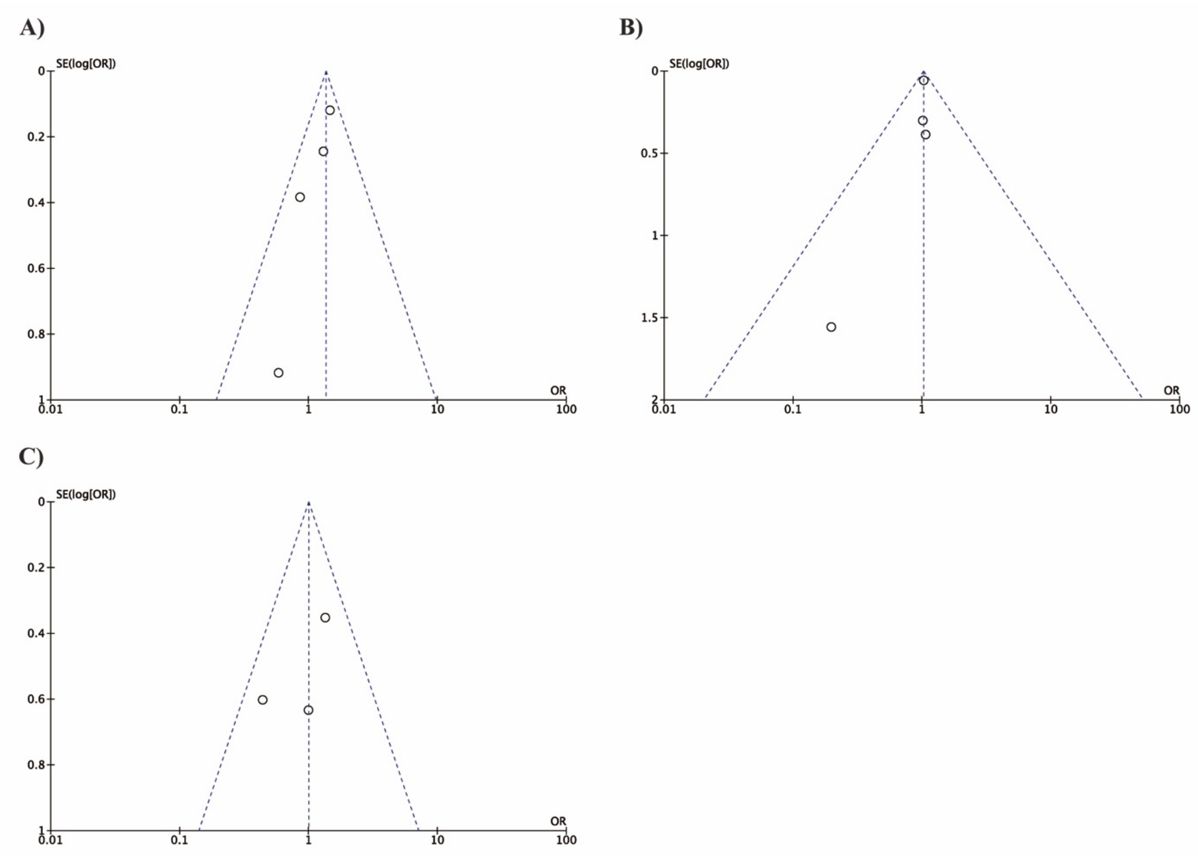

Supplementary Fig. S8. Funnel plot showing publication bias for the effect of different P2Y12 inhibitors on frequencies of Thrombolysis in Myocardial Infarction (TIMI) major bleeding in patients with acute coronary syndrome

(A) prasugrel versus clopidogrel. (B) ticagrelor versus clopidogrel. C) cangrelor versus clopidogrel.

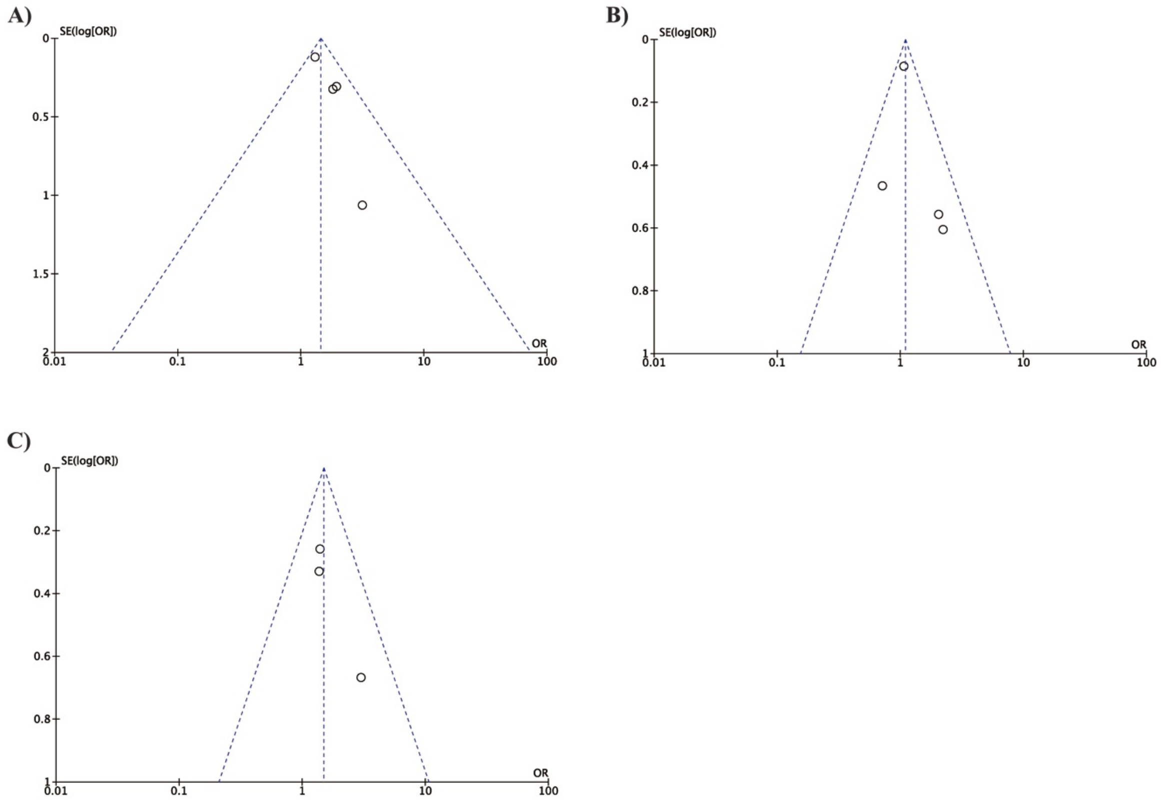

Supplementary Fig. S9. Funnel plot showing publication bias for the effect of different $P2Y_{12}$ inhibitors on frequencies of Thrombolysis in Myocardial Infarction (TIMI) minor bleeding in patients with acute coronary syndrome

(A) prasugrel versus clopidogrel. (B) ticagrelor versus clopidogrel. (C) cangrelor versus clopidogrel.

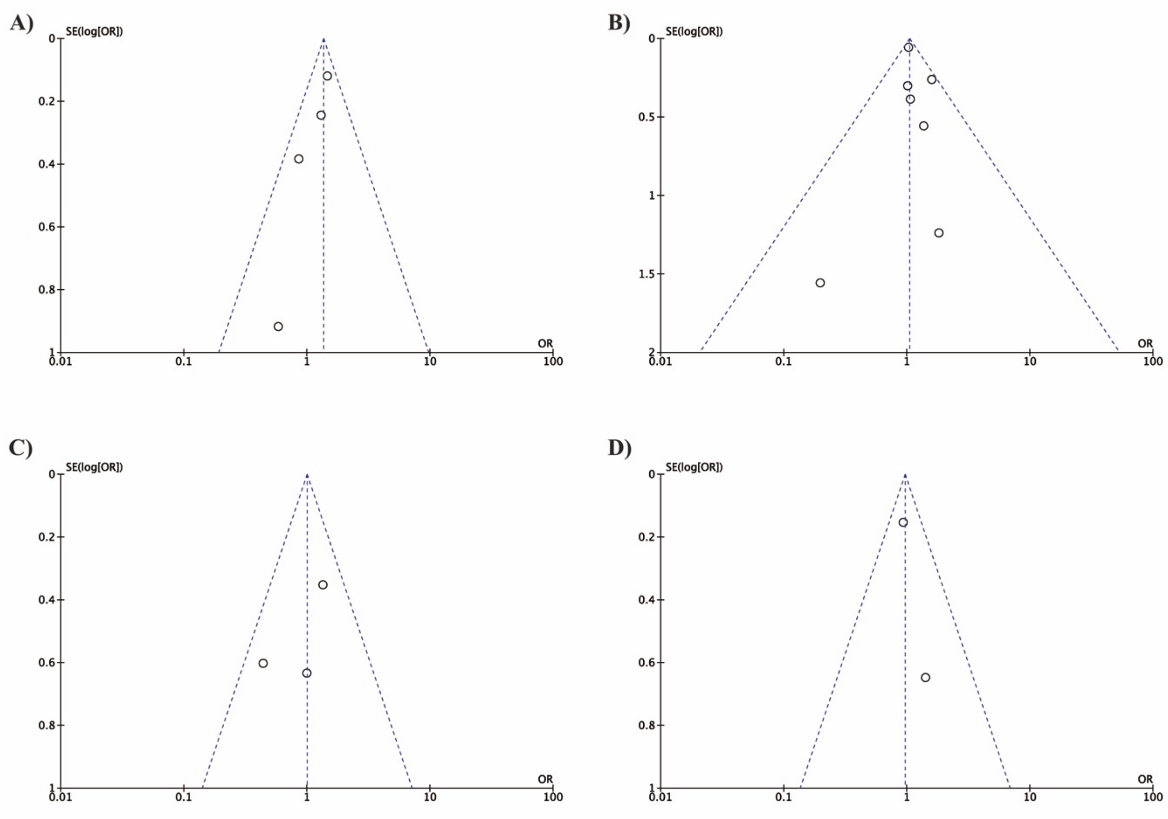

Supplementary Fig. S10. Funnel plot showing publication bias for the effect of different P2Y12 inhibitors on frequencies of all major bleeding in patients with acute coronary syndrome

(A) prasugrel versus clopidogrel. (B) ticagrelor versus clopidogrel. (C) cangrelor versus clopidogrel. D) prasugrel versus ticagrelor.

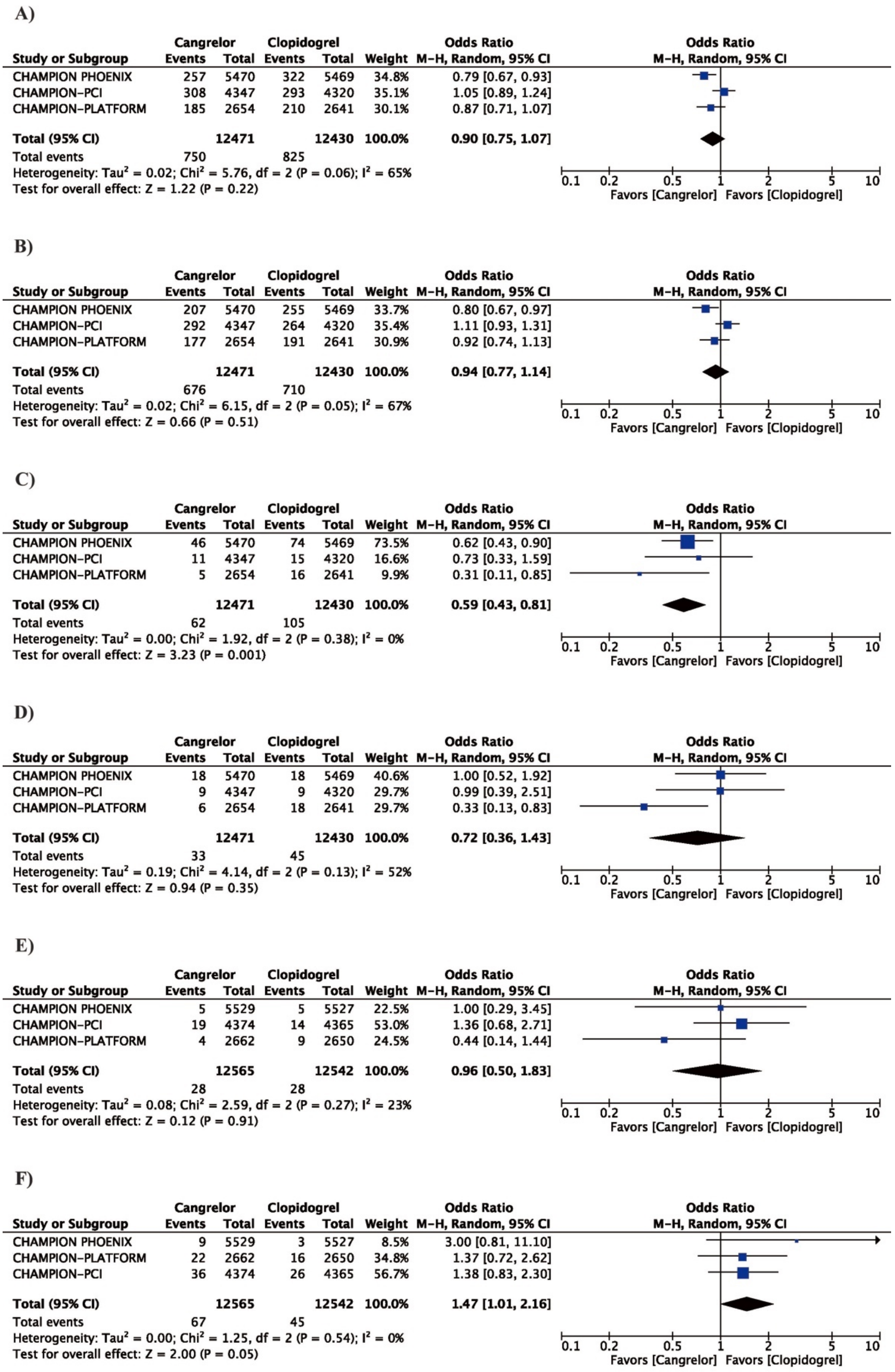

Supplementary Fig. S11. Forest plots of the effects of cangrelor on A). MACE; B). Myocardial infarction; C). Definite or probable stent thrombosis; D). All-cause mortality; E). TIMI major bleeding; F). TIMI minor bleeding. Square markers indicate odds ratios for clinical outcomes comparing different cangrelor to clopidogrel. The horizontal lines indicate 95% confidence intervals. MACE = major adverse cardiovascular events.
